# Geochronological and Taxonomic Revisions of the Middle Eocene Whistler Squat Quarry (Devil’s Graveyard Formation, Texas) and Implications for the Early Uintan in Trans-Pecos Texas

**DOI:** 10.1371/journal.pone.0101516

**Published:** 2014-07-02

**Authors:** Christopher J. Campisano, E. Christopher Kirk, K. E. Beth Townsend, Alan L. Deino

**Affiliations:** 1 Institute of Human Origins, Arizona State University, Tempe, Arizona, United States of America; 2 School of Human Evolution and Social Change, Arizona State University, Tempe, Arizona, United States of America; 3 Department of Anthropology, University of Texas at Austin, Austin, Texas, United States of America; 4 Vertebrate Paleontology Laboratory, Jackson School of Geosciences, University of Texas at Austin, Austin, Texas, United States of America; 5 Department of Anatomy, Arizona College of Osteopathic Medicine, Midwestern University, Glendale, Arizona, United States of America; 6 Berkeley Geochronology Center, Berkeley, California, United States of America; Raymond M. Alf Museum of Paleontology, United States of America

## Abstract

The Whistler Squat Quarry (TMM 41372) of the lower Devil’s Graveyard Formation in Trans-Pecos Texas is a middle Eocene fossil locality attributed to Uintan biochronological zone Ui1b. Specimens from the Whistler Squat Quarry were collected immediately above a volcanic tuff with prior K/Ar ages ranging from ∼47–50 Ma and below a tuff previously dated to ∼44 Ma. New ^40^Ar/^39^Ar analyses of both of the original tuff samples provide statistically indistinguishable ages of 44.88±0.04 Ma for the lower tuff and 45.04±0.10 Ma for the upper tuff. These dates are compatible with magnetically reversed sediments at the site attributable to C20r (43.505–45.942 Ma) and a stratigraphic position above a basalt dated to 46.80 Ma. Our reanalysis of mammalian specimens from the Whistler Squat Quarry and a stratigraphically equivalent locality significantly revises their faunal lists, confirms the early Uintan designation for the sites, and highlights several biogeographic and biochronological differences when compared to stratotypes in the Bridger and Uinta Formations. Previous suggestions of regional endemism in the early Uintan are supported by the recognition of six endemic taxa (26% of mammalian taxa) from the Whistler Squat Quarry alone, including three new taxa. The revised faunal list for the Whistler Squat Quarry also extends the biostratigraphic ranges of nine non-endemic mammalian taxa to Ui1b.

## Introduction

**Figure 1:**
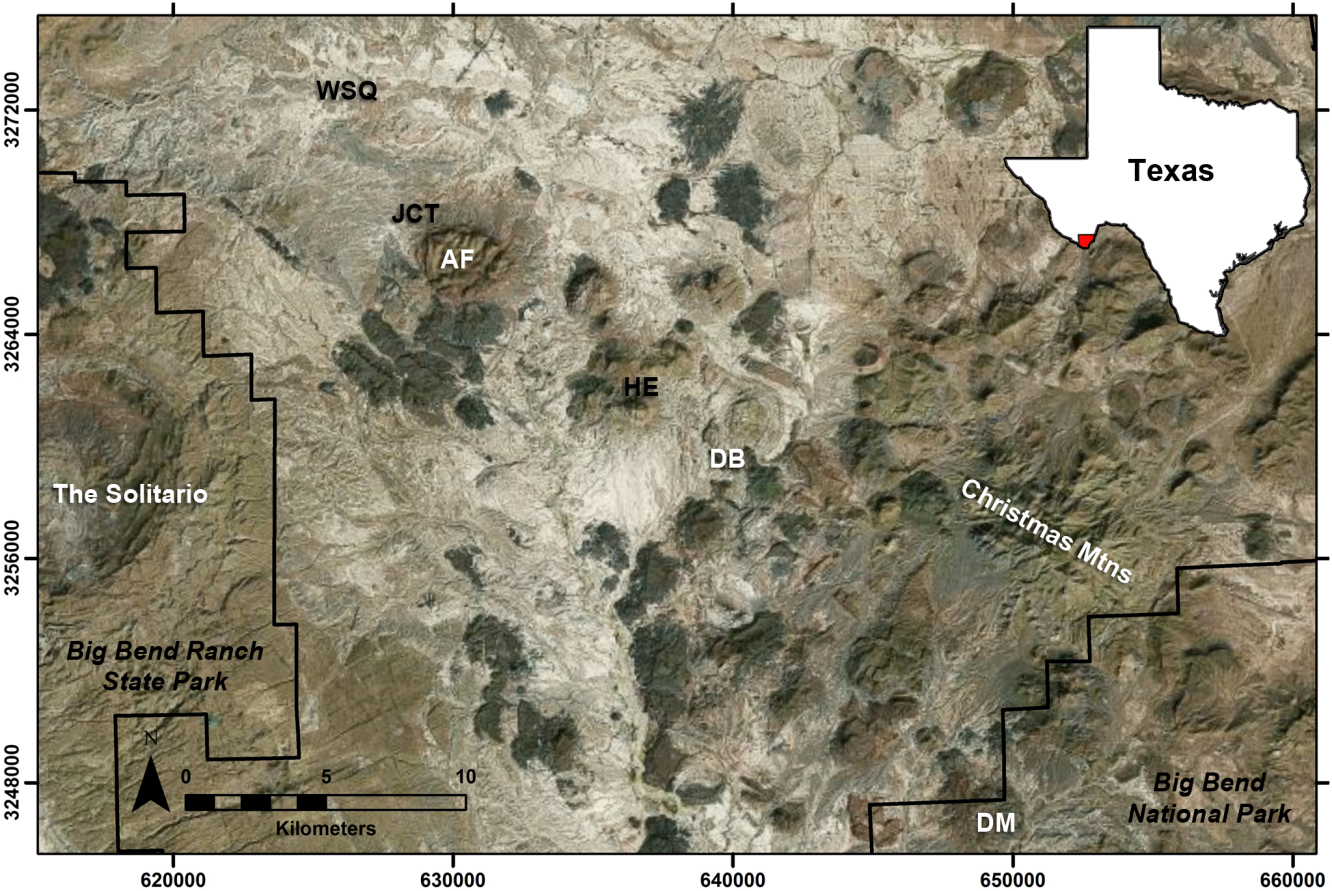
Satellite image of the Devil’s Graveyard Formation study area and surrounding region. WSQ = Whistler Squat Quarry; JCT = Junction locality; AF = Agua Fria Mountain; HE = Hen Egg Mountain; DB = Devil’s Graveyard basalt; DM = Dogie Mountain.

The transition between the Bridgerian and Uintan North American Land Mammal ages (NALMAs) remains one of the most problematic biostratigraphic intervals in the Eocene. Although several well-documented faunal assemblages are known from the late Bridgerian and early Uintan, few continuous fossiliferous sequences span the Bridgerian–Uintan transition [1], [2]. Furthermore, patterns of regional endemism complicate attempts to make precise biostratigraphic correlations between localities [3], [4]. The Devil’s Graveyard Formation (DGF) of Trans-Pecos Texas (Figure 1) is particularly relevant for understanding the tempo and mode of mammalian evolution during the early Uintan because it preserves abundant middle Eocene faunal assemblages stratified within volcaniclastic sediments dateable by both radiometric and paleomagnetic techniques.

**Figure 2:**
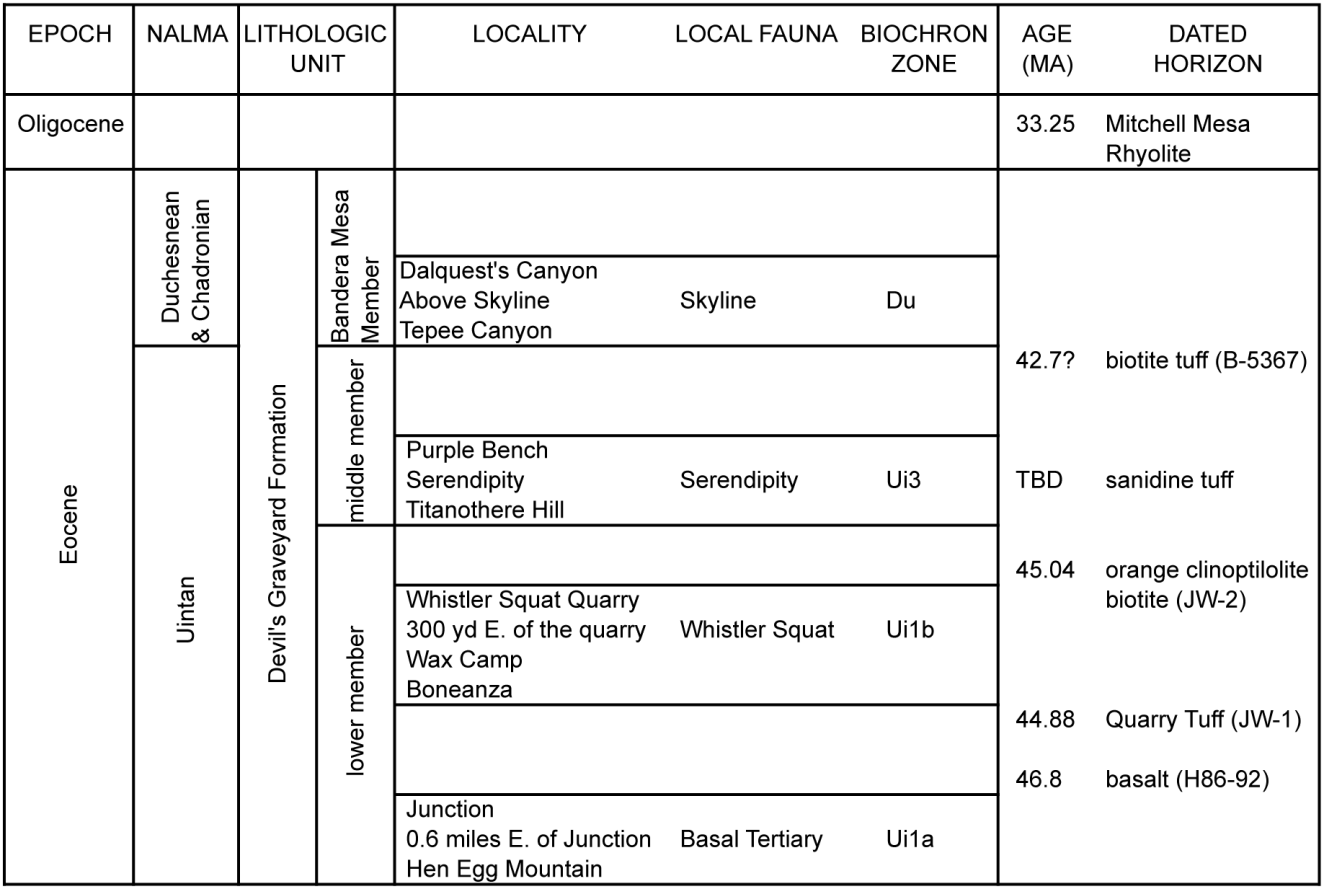
Lithostratigraphy, biochronology and radiometric ages of the Devil’s Graveyard Formation in the Agua Fria and Hen Egg Mountain region. Sample numbers for dated horizons are in parentheses. The 1980s K/Ar age for sample B-5367 of 42.7±1.6 Ma [8], [14] has been assumed to be erroneously old by others (e.g., [96]) and is being redated. Locality numbers: Hen Egg Mountain (TMM 42028, TMM 42287); 0.6 miles east of Junction (TMM 41444); Junction (TMM 41443); Boneanza (TMM 41747); Wax Camp (TMM 41576); Whistler Squat Quarry (TMM 41372); 300 yd east of Whistler Squat Quarry (TMM 41466); Titanothere Hill (TMM 41723); Serendipity (41745); Purple Bench (TMM 41672); Tepee Canyon (TMM 41578); Above Skyline (TMM 41580); Dalquest’s Canyon (TMM 41715).

Fossil materials assigned to the Whistler Squat local fauna of the DGF were collected by University of Texas field parties under the direction of J.A. Wilson from 1970–1974 and are curated at the Jackson School of Geosciences Vertebrate Paleontology Laboratory at the University of Texas at Austin. As defined by Walton [5], [6], the Whistler Squat local fauna consists of material from four localities recovered from an equivalent stratigraphic sequence in the DGF: (1) TMM 41372 (“Whistler Squat Quarry”); (2) TMM 41466, described as “located about 300 yards east of” TMM 41372 ([7]: p. 354) although specimen notes document that these fossils were collected over a large area adjacent to the quarry as opposed to a discrete locality (E.C.K., pers. obs.); (3) TMM 41576 (“Wax Camp”), located ∼1.5 km west of TMM 41372; and (4) TMM 41747 (“Boneanza”), located ∼750 m northeast of TMM 41372. This study focuses only on specimens from the Whistler Squat Quarry (*n* = 815) and TMM 41466 (*n* = 16), which are derived from the same stratum (M.S. Stevens, pers. comm., May, 2013) and together comprise 84% of the specimens in the Whistler Squat local fauna. Fossils from Wax Camp and Boneanza are not considered here because the exact stratigraphic provenance of these two localities is not as well documented. Wilson [7] originally grouped fossils from the “basal Tertiary conglomerate” of the lowermost DGF with those of the stratigraphically higher Whistler Squat Quarry level as comprising the Whistler Squat local fauna. However, he also noted that further collecting might favor the allocation of localities in these two stratigraphic intervals to different local faunas [7]. Walton [5], [6] subsequently recognized a Basal Tertiary local fauna for the older DGF localities (Figure 2) based on key differences between these two stratigraphic intervals, such as the first appearance of *Amynodon* in the Whistler Squat Quarry. Reviews by Robinson et al. [1] and Gunnell et al. [2] also recognize a biostratigraphic difference between these two intervals within the lower member of the DGF, but do not use the term Basal Tertiary local fauna for the older material.

Robinson et al. [1] erected the Uintan biochron Ui1 to accommodate those assemblages that represent a transitional interval between Bridgerian biochron Br3 and the early Uintan faunal assemblages typified by Uinta B localities in Utah and assigned to Uintan biochron Ui2. Furthermore, Robinson et al. [1] assigned the Whistler Squat local fauna of the DGF to either Ui1 or Ui2. Gunnell et al. [2] subsequently subdivided Ui1 into biochrons Ui1a and Ui1b based on differences in the number of Bridgerian holdover taxa, the diversity of selenodont artiodactyls, and the first appearance of *Amynodon*. The Turtle Bluff Member ( = Bridger E) of the Bridger Formation was selected as the statotype for the Ui1a biochron, but due to the lack of high resolution lithostratigraphic data associated with Ui1b faunal assemblages, no stratotype was assigned to biochron Ui1b. Instead, the biochron is associated with a half-dozen well-documented faunal assemblages including the Whistler Squat local fauna of the DGF.

In this study, we revise the radiometric age and reassess the mammalian specimens from two localities of the Whistler Squat local fauna for the first time in nearly three decades. Our geochronological results provide a tightly constrained age for the Whistler Squat localities, and our reanalysis of fossil specimens significantly revises the Whistler Squat faunal list. These results have important implications for early Uintan biochronology and for documenting regional patterns of endemism in the middle Eocene of North America.

Abbreviations: **TMM**, Jackson School of Geosciences Vertebrate Paleontology Laboratory at the University of Texas at Austin, formerly “Texas Memorial Museum”, Austin, Texas, USA; **USNM**, Smithsonian Institution National Museum of Natural History, Washington, D.C., USA; **YPM**, Yale University Peabody Museum of Natural History, New Haven, Connecticut, USA; **p**, mandibular premolar; **P**, maxillary premolar; **m**, mandibular molar; **M**, maxillary molar; **Ma**, mega-annum.

### Geological Context

The Devil’s Graveyard Formation (DGF), part of the Buck Hill Group, is composed of more than 472 meters of middle Eocene to early Oligocene continental volcaniclastic sediments located in the south-central part of the Trans-Pecos volcanic field of West Texas [8]. The DGF is exposed principally at the western edge of Brewster County in the Agua Fria region between Big Bend National Park and Big Bend Ranch State Park (Figure 1). Additional exposures attributed to the DGF have been mapped to the southeast of Agua Fria in the vicinity of Hen Egg Mountain and Dogie Mountain [9], [10] (Figure 1). In its type area, the DGF unconformably overlies late Cretaceous marine sediments, and is capped by the ∼33.25 Ma Mitchell Mesa Rhyolite [8], [11]. Stevens et al. [8] divided the DGF into three units based principally on local disconformities, the informal lower and middle members and the formal Bandera Mesa Member (Figure 2). Fossil localities in the basal portion of the lower member comprise the early Uintan Basal Tertiary local fauna, those in the upper portion of the lower member comprise the early Uintan Whistler Squat local fauna, those from the middle member comprise the late Uintan Serendipity local fauna, and those from the Bandera Mesa Member comprise the Duchesnean Skyline local fauna [5], [6], [7], [8], [12] (Figure 2).

**Figure 3:**
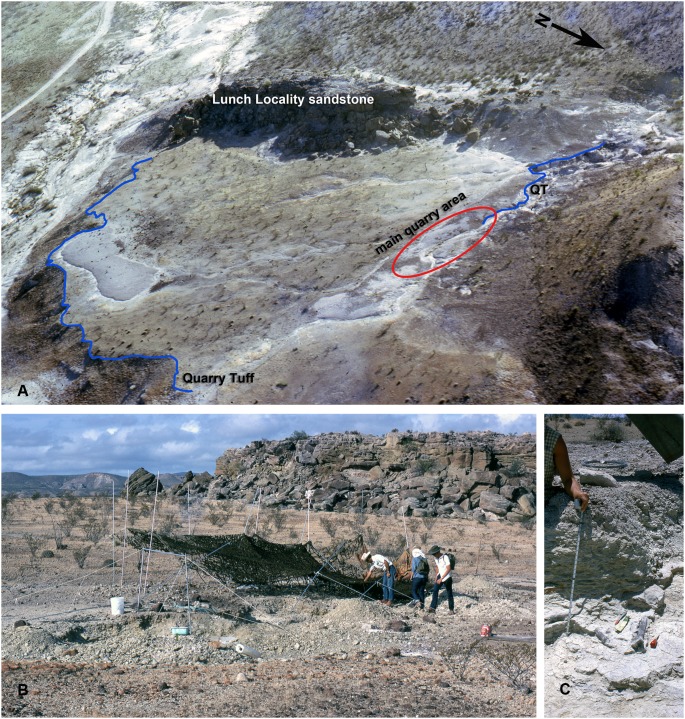
Photos of the Whistler Squat Quarry locality. From the archives of the Vertebrate Paleontology Laboratory and courtesy of Sarah Wilson. **A**, 1970 aerial view of the locality prior to any significant quarrying with key features marked; **B**, excavation at the quarry, July 1971; **C**, close-up of the ∼20 cm fossiliferous horizon immediately overlying the Quarry Tuff, August 1971.

Wilson [7] described the main fossiliferous unit of the Whistler Squat Quarry as a weakly calcareously cemented bentonitic clay-pebble conglomerate with sanidine and biotite grains. This unit ranges up to 55 cm thick and is likely a fluvial channel remnant because it grades upward into cross-bedded sands and preserves variable degrees of water-worn bone (J.A. Wilson, 1970 field notes, TMM). At the Whistler Squat Quarry a 15–50 cm thick calcareous tuff, referred to as the “Quarry Tuff” by Walton [5], directly underlies the fossiliferous unit [7], [8] (Figure 3). The Quarry Tuff was described as a welded tuff discontinuously exposed from the southwest to northeast corners of the type area with a distinctive fractured, yellow-stained surface in outcrop [5], [8]. At the Whistler Squat Quarry, the resistant tuff forms a low bluff that the fossiliferous unit rests upon, with several areas excavated across the exposed platform (Figure 3). The tuff and clay-pebble conglomerate grade laterally into a tuffaceous limestone, reported to represent a depositional environment close to the edge of a small lake [7]. In some locations, an additional 10–90 cm of mottled yellow and purple bentonite that is sparsely fossiliferous separates the tuff from the channel deposit (J.A. Wilson, 1971 field notes, TMM). Samples JW-1 and 72-WS33-2 were collected for dating from the Quarry Tuff at the Whistler Squat Quarry. The Lunch Locality sandstone, 5–6 m above the Quarry Tuff at the Whistler Squat Quarry, is a ledge forming sandstone on the west side of the excavated areas (Figure 3) that along with several other localized channel sands at the same approximate stratigraphic level have been referred to as the “Lunch Complex” [5], [8]. Approximately 1.5 km south of the Whistler Squat Quarry, sample JW-2 was collected from a biotite-rich ash in the lowest part of the Lunch Complex associated with the “orange clinoptilolite” (a product of altered volcanic glass) ([5], J.A. Wilson, 1971 field notes, TMM). Thus, the two samples from the Quarry Tuff and JW-2 bracket the Whistler Squat Quarry and TMM 41466, although the Quarry Tuff is more directly associated with the fossil fauna in both geographic and stratigraphic context.

Samples JW-1, 72-WS33-2, and JW-2 were first dated in the 1970s by F. McDowell at the University of Texas at Austin. Age results have been reported in several publications (e.g., [8], [13], [14]), but the complete analytical details and data reduction have never been published. Sanidine separates from JW-1 and plagioclase separates from 72-WS33-2 yielded K/Ar ages of 46.9±1.0 Ma and 49.7±1.2 Ma, respectively. Biotite separates from JW-2 yielded a K/Ar age of 43.9±0.7 Ma. This study takes advantage of the significant technological advances over the last several decades to provide single-crystal ^40^Ar/^39^Ar analyses of samples JW-1 and JW-2 and the Whistler Squat Quarry assemblage.

## Materials and Methods

### Geochronology

Tephra samples JW-1 and JW-2 were collected by J. Wilson in 1970 and 1971, respectively. Both samples are distal tuffs. JW-1 is well indurated, whereas JW-2 is soft and friable. In thin section, both samples are matrix supported by a groundmass of fine clay that is likely the alteration product of volcanic glass shards. The phenocryst population includes quartz and potassium feldspar of approximately equal amounts, with subordinate plagioclase and biotite and sparse lithic fragments (J.W. McDowell, pers. comm., March, 2013). Sample JW-1 appears to be an undisturbed ash-fall deposit, confirming the original description of the Quarry Tuff as a primary tephra deposit. The JW-2 thin section indicates some degree of winnowing of ash compared to JW-1, but we still interpret it as an unreworked fallout tuff as opposed to a “tuffaceous sediment” as noted in Henry et al. [14].

Sample preparation was principally conducted at the University of Texas at Austin by J.W. McDowell in the 1970s. Both samples were processed through a jaw crusher and disk mill, the latter at a close adjustment to liberate crystals from ground mass. Samples were screened to retain the 60–80 mesh (180–250 micron) size fraction. They were then washed and passed through a magnetic separator, in the case of JW-1 to eliminate all magnetic particles and for JW-2 to roughly define the range of magnetic susceptibility for the biotite. Sample JW-2 was processed through bromoform with a specific gravity of 2.86 and further refined by ultrasonic cleaning and sieving, followed by more careful magnetic separation. The finished separate was checked by x-ray diffraction for crystalinity and chlorite content. For JW-1, following a leach in 10% hydroflouric acid and sonification to remove fines, the feldspar of JW-1 was separated with bromoform adjusted with acetone to a specific gravity of approximately 2.60 or lighter to eliminate quartz and then at 2.56 to eliminate groundmass. Finally, X-ray diffraction was used to assess residual quartz content. The original feldspar separates from JW-1 and biotite separates from JW-2 were subsequently re-screened for the 40–60 mesh (250–425 micron) size fraction, with the most euhedral and unaltered crystals hand-picked under a stereomicroscope at Arizona State University.

The crystal concentrates were irradiated in a single batch for 50 hours in the Cd-lined, in-core CLICIT facility of the Oregon State University TRIGA reactor. Sanidine from the Fish Canyon Tuff of Colorado was used as the neutron fluence monitor, with an astronomically calibrated reference age of 28.201±0.046 Ma [15]. Standards and unknowns were placed in 2 mm deep wells in circular configurations on 18 mm diameter aluminum disks, with standards placed strategically so that the lateral neutron flux gradients across the disk could be evaluated. Planar regressions were fit to the standard data, and the ^40^Ar/^39^Ar neutron fluence parameter, *J*, interpolated for the unknowns. *J*’s are measured independently for each machine and approach, and in this case differ by ∼1% between the systems. Uncertainties in *J* are estimated at ∼0.05% for the single-crystal total-fusion (SCTF) data set on the MAP machine, and ∼0.2% for the single-crystal incremental-heating (SCIH) work on the Noblesse, based on Monte Carlo error analysis of the planar regressions [16].

All ^40^Ar/^39^Ar analytical work was performed at the Berkeley Geochronology Center (BGC). Argon extractions from the irradiated material were performed on two separate systems. The SCTF work utilized a partially defocused CO_2_ laser beam delivering ∼5–8 Watts of power to rapidly fuse individual feldspars over an interval of ∼6 seconds. Released gasses were exposed for several minutes to an approximately 143 K cryo-surface to trap H_2_0, and to SAES getters to remove reactive compounds (CO, CO_2_, N_2_, O_2_, and H_2_). After approximately three minutes of cleanup, the gas was admitted to an MAP 215-50 mass spectrometer. Five argon isotopes were measured by peak hopping on a single analog multiplier over a period of approximately 30 minutes. Measured isotope abundances were corrected for extraction-line blanks. A value of 295.5 was used for the atmospheric ^40^Ar/^36^Ar ratio [17] for the purposes of routine measurement of mass spectrometer discrimination using air aliquots, and correction for atmospheric argon in the ^40^Ar/^39^Ar age calculation. Additional details of the total-fusion feldspar dating methodology as applied at BGC are provided elsewhere [16], [18], [19], [20].

The SCIH work was performed on a completely separate extraction line and mass spectrometer combination. Laser heating was achieved using a CO_2_ laser fitted with a beam-shaping lens that generates a flat energy profile of variable diameter. Individual grains of biotite were heated for ∼30 seconds at progressively increasing power levels until fusion was achieved (5–8 steps). After a cleanup interval of several minutes analogous to that described above, the argon isotopes were measured by ion counting on a 5-collector Noblesse mass spectrometer over a period of about eight minutes. Isotopes ^40^Ar, ^39^Ar, ^37^Ar, and ^36^Ar were detected simultaneously on separate ion counters, interspersed with a brief peak hop to bring ^38^Ar onto an ion counter for measurement. Count rates were kept below 200 kcps to minimize dead-time corrections. Detector intercalibrations were performed with periodic measurement of air argon (^40^Ar/^36^Ar by comparison of simultaneous measurement to the expected air ratio of 295.5, and ^40^Ar/^39^Ar, ^40^Ar/^38^Ar, and ^40^Ar/^37^Ar by repeated measurement of ^40^Ar on relevant detectors). Measurement of the neutron flux standard (FC Sanidine) was also performed by SCIH on the same machine using the same protocols as the unknown.

### Paleontology

This study examined mammalian specimens from localities TMM 41372 (Whistler Squat Quarry) and TMM 41466 (representing the same stratigraphic interval as the Whistler Squat Quarry) housed at the Jackson School of Geosciences Vertebrate Paleontology Laboratory at the University of Texas at Austin, a publicly accessible paleontological repository. No permits were required for the described study, which complied with all relevant regulations. All fossil specimens were collected in the 1970s on private land (Agua Fria Ranch) with permission of the landowners (M. Richmond and J.H. Burton) and lessees (B.P. and S. McKinney). All examined specimens are listed in the Systematic Paleontology section below. Some of these specimens were previously used to generate a faunal list for the “Whistler Squat quarry and equivalent localities” by Wilson ([7]: p. 371). No other systematic re-assessment of the mammalian fossil sample from these two localities has been undertaken in the intervening 27 years. Although some specimens from TMM 41372 and TMM 41466 have been included in earlier descriptions of the DGF mammalian fauna (see below), formal examination and comparison of other specimens used in this analysis have not been completed previously.

## Results

### Geochronology

**Figure 4:**
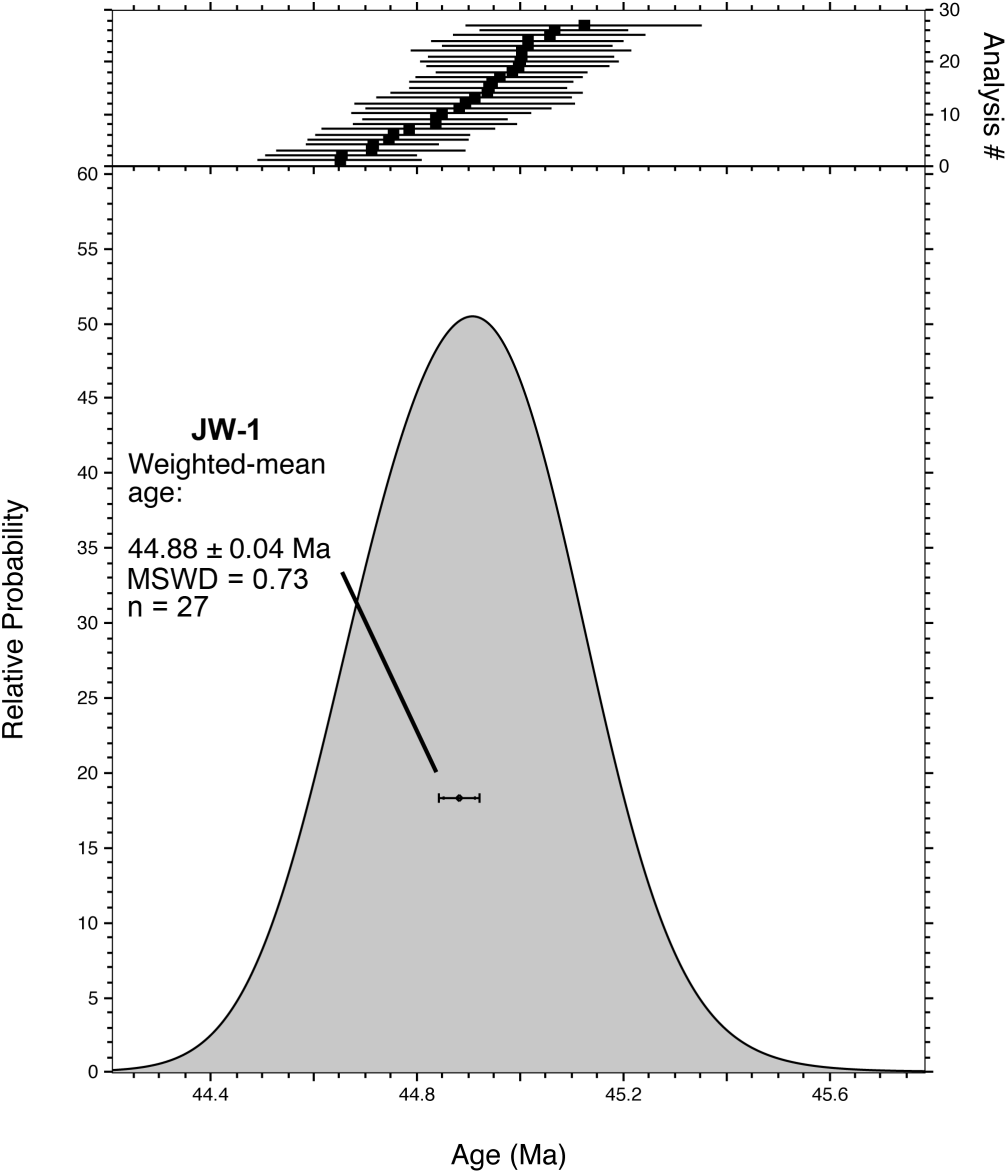
^40^Ar/^39^Ar age distribution of JW-1 phenocrysts. Age-probability density function and weighted-mean age of the ^40^Ar/^39^Ar single-crystal total-fusion (SCTF) analyses of individual sanidine phenocrysts from sample JW-1. Complete analytical data provided in Table S1.

Single-crystal total-fusion ^40^Ar/^39^Ar dating of 27 sanidine phenocrysts from JW-1 yielded a simple unimodal distribution of ages (Figure 4) and a weighted mean age of 44.88±0.04 Ma (1σ error including error in *J*, MSWD = 0.73) (Table 1, Table S1). This high-precision result (± ∼0.08%) serves as an excellent chronostratigraphic tie-point for the section.

**Table 1:** Summary single-crystal total-fusion ^40^Ar/^39^Ar analytical results for JW-1 sanidine phenocrysts.

Lab ID#	^39^Ar Mol x10^−14^	%^40^Ar*	Ca/K	Age ±1σ (Ma)^1^
25958-01	5.25	99.0	0.0249	44.90±0.20
25958-03	9.22	98.9	0.0169	44.95±0.15
25958-05	7.09	99.1	0.0240	44.88±0.17
25958-09	10.96	99.1	0.0258	44.84±0.13
25958-10	10.16	99.6	0.0219	45.07±0.14
25958-11	6.92	99.3	0.0248	44.91±0.18
25958-12	7.84	99.6	0.0229	45.02±0.16
25958-13	10.50	98.8	0.0333	44.72±0.12
25958-14	7.08	99.3	0.0295	45.00±0.17
25958-15	8.04	95.9	0.0270	44.84±0.15
25958-16	9.12	98.7	0.0252	44.76±0.14
25958-17	6.04	99.0	0.0338	45.00±0.18
25958-18	7.36	98.8	0.0178	44.85±0.16
25958-19	6.53	98.6	0.0844	45.01±0.17
25958-20	6.52	98.1	0.0440	44.94±0.18
25958-21	9.00	99.1	0.0290	44.99±0.14
25958-22	7.34	99.6	0.0338	44.96±0.15
25958-23	8.29	98.7	0.0284	44.65±0.15
25958-24	8.15	99.2	0.0327	44.94±0.15
25958-25	7.79	99.1	0.0227	44.75±0.15
25958-26	5.16	98.8	0.0303	45.13±0.22
25958-27	6.97	99.0	0.0178	45.06±0.18
25958-28	7.05	99.0	0.0218	45.02±0.18
25958-29	6.74	98.8	0.0291	44.71±0.17
25958-30	5.19	98.9	0.0266	45.01±0.20
25958-31	8.43	98.8	0.0295	44.66±0.14
25958-32	7.19	98.7	0.0260	44.79±0.16
Weighted Mean				44.88±0.04
MSWD				0.73

1 Includes error in *J*, the neutron fluence parameter.

MSWD = Mean Square Weighted Deviation.

Complete analytical data provided in Table S1.

**Figure 5:**
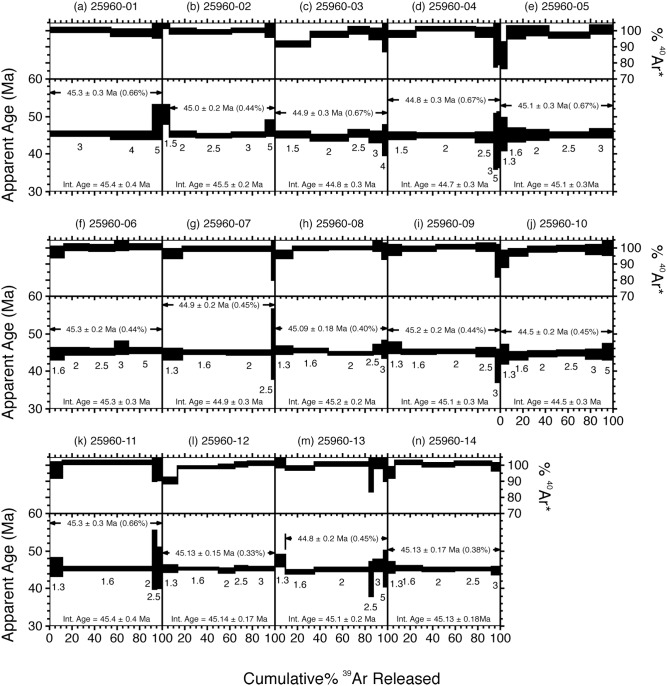
^40^Ar/^39^Ar incremental heating spectra of JW-2 phenocrysts. Spectra derived from ^40^Ar/^39^Ar single-crystal incremental-heating (SCIH) analyses of individual biotite phenocrysts from sample JW-2. Excludes steps yielding less than 2% of the total ^39^Ar released. Complete analytical data provided in Table S1.

**Figure 6:**
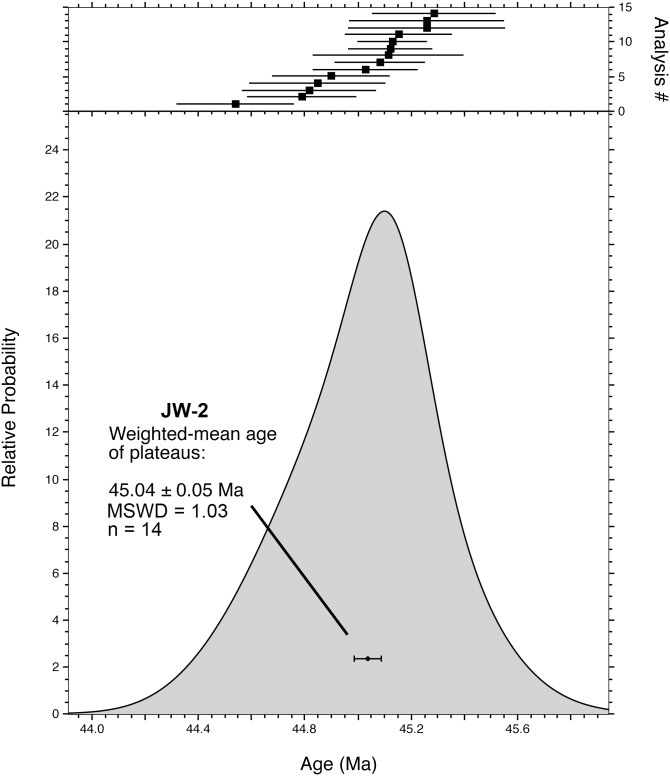
^40^Ar/^39^Ar age distribution of JW-2 phenocrysts. Age-probability density function and weighted-mean age of the plateau ages from the single-grain biotite ^40^Ar/^39^Ar SCIH analyses. Complete analytical data provided in Table S1.

Fourteen SCIH ^40^Ar/^39^Ar dating experiments on single biotite grains from JW-2 are illustrated as incremental release spectra in Figure 5, with analytical data provided in Table 2 and Table S1. Note that these data sets exclude steps yielding less than 2% of the total ^39^Ar released. Every experiment yielded a plateau (defined by consecutive steps in which there is greater than a 95% chance that the Mean Square of Weighted Deviates [‘MSWD’] of ages is accounted by measurement error alone) encompassing >90% of the total ^39^Ar released. Further, each experiment revealed high radiogenic content vs. atmospheric contamination, registering >90% radiogenic argon (^40^Ar*) in all but a few of the earliest steps. Thus, the internal systematics of the individual biotite spectra suggests that they are fresh, undisturbed grains that potentially yield accurate geological information. The population distribution of the plateau ages for JW-2 yielded a simple unimodal distribution (Figure 6), with a weighted mean of 45.04±0.10 Ma (1σ error including error in *J*, MSWD = 1.03, *n* = 14) (Table 2).

**Table 2:** Summary single-crystal incremental-heating ^40^Ar/^39^Ar analytical results for JW-2 biotite phenocrysts.

	Apparent-Age Plateau				Integrated
Run ID	Age ± 1σ (Ma)^1^	MSWD	*n/n_tot_*	%^39^Ar	Age ± 1σ (Ma)^2^
25960-01	45.3±0.3	1.2	3/3	100.0	45.4±0.4
25960-02	45.00±0.18	1.2	4/5	93.2	45.5±0.2
25960-03	44.9±0.2	1.1	5/5	100.0	44.8±0.3
25960-04	44.8±0.2	0.2	5/5	100.0	44.7±0.3
25960-05	45.1±0.3	0.1	5/5	100.0	45.1±0.3
25960-06	45.3±0.2	0.6	5/5	100.0	45.3±0.3
25960-07	44.9±0.2	0.2	4/4	100.0	44.9±0.3
25960-08	45.09±0.16	1.2	5/5	100.0	45.2±0.2
25960-09	45.2±0.2	1.3	5/5	100.0	45.1±0.3
25960-10	44.5±0.2	0.4	6/6	100.0	44.5±0.3
25960-11	45.3±0.3	0.1	4/4	100.0	45.4±0.4
25960-12	45.13±0.12	0.7	5/5	100.0	45.14±0.17
25960-13	44.8±0.19	1.9	5/6	90.7	45.1±0.2
25960-14	45.13±0.15	0.5	5/5	100.0	45.13±0.18
Mean	45.04±0.10^2^				45.1±0.11

1 Excludes error in *J*, the neutron fluence parameter, except as noted.

2 Includes error in *J.*

*n/n_tot_* = steps used to calculate age/steps yielding >2% of the total 39Ar released.

MSWD = Mean Square Weighted Deviation.

Complete analytical data provided in Table S1.

The biotite mean plateau age for sample JW-2 is statistically indistinguishable at the 95% confidence level from the sanidine age of the stratigraphically lower sample JW-1. Of these two sample ages, the result that best defines the age of the fossiliferous horizon is clearly the sanidine determination from JW-1 of 44.88±0.04 Ma. This result is not only more analytically precise, but sanidine is potentially more geologically accurate due to the greater susceptibility to alteration of biotite. The chronostratigraphic tie point provided by the sanidine date is also convenient because the dated tuff lies immediately below the fossil horizon.

### Systematic Paleontology

Order DIDELPHIMORPHIA Gill, 1872 [21].

Family HERPETOTHERIIDAE Trouessart, 1879 [22].

Genus *HERPETOTHERIUM* Cope, 1873 [23].

HERPETOTHERIUM sp.

Specimens: TMM 41372-239, -403.

**Figure 7:**
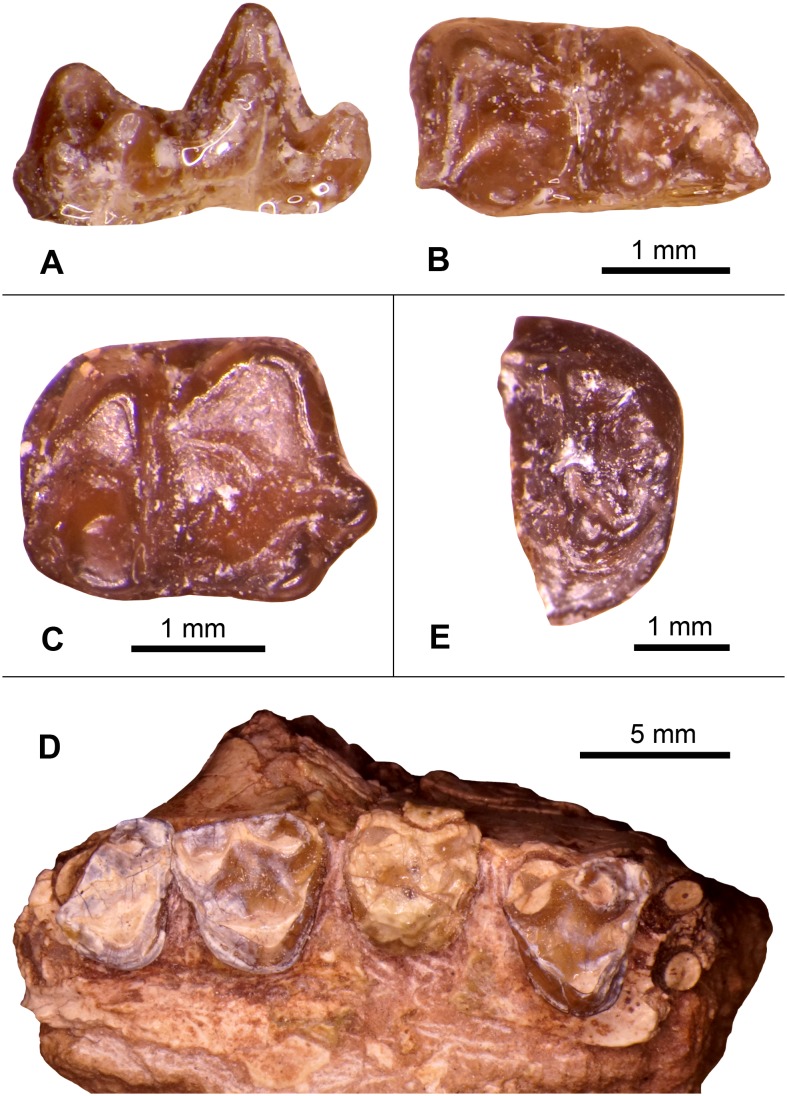
Select dental specimens from the Whistler Squat local fauna. **A–B**, *Herpetotherium* sp., left m1/2 (TMM 41372-403), lingual view (A) and occlusal view (B); **C**, *Scenopagus* cf. *S. edenensis*, right mandibular molar (TMM 41372-118), occlusal view; **D**, *Microsyops annectens*, partial right maxilla with P4-M3 (TMM 41466-7), occlusal view; **E**, *Ourayia uintensis*, right m2 trigonid (TMM 41372-301), occlusal view.

These two isolated lower molars from the Whistler Squat Quarry were included by West [24] in an assemblage of isolated teeth that he attributed to *Herpetotherium marsupium* (Figure 7A–B). *Herpetotherium* is known from many North American Eocene localities and ranges from the Wasatchian through the Duchesnean [25]. Species level designations for *Herpetotherium* are primarily based on characters of the upper molars, which are not represented in the Whistler Squat Quarry sample. Lower molars of the known species of *Herpetotherium* may differ in size, but are very similar in occlusal morphology [26], [27]. Accordingly, West’s [24] attribution of the Whistler Squat Quarry specimens to *H. marsupium* was based on the morphology of upper molars recovered from Ui1a localities that are stratigraphically lower in the Devil’s Graveyard Formation (i.e., TMM 41443 “Junction” and TMM 41444 “0.6 miles east of Junction”; Figure 2). TMM 41372-403 very closely resembles the m2 of the holotype mandible of *H. marsupium* (YPM 13518; [28]) in both size and morphology. The Whistler Squat Quarry *Herpetotherium* lower molars also compare favorably in size with lower molars attributed to *H. marsupium* from the Uintan Swift Creek local fauna [29] and with *Herpetotherium* cf. *H. marsupium* from the Duchesnean Lac Pelletier local fauna [26], [27]. Nevertheless, TMM 41372-403 is also similar in size to lower molars attributed to the Bridgerian-Duchesnean species *H. knighti* and TMM 41372-239 is similar in size to lower molars attributed to the Wasatchian-Duchesnean species *H. innominatum* [2], [24], [26], [30], [31]. Given this size overlap with multiple Uintan species of *Herpetotherium* and the lack of associated upper molars from the Whistler Squat Quarry that might be used for species identification, we refer TMM 41372-239 and TMM 41372-403 to *Herpetotherium* sp.

Both of the Whistler Squat Quarry specimens are either first or second lower molars. The first through third mandibular molars of *Herpetotherium* are nearly identical in cusp morphology and differ primarily in their size and the relative proportions of the trigonid and talonid [26]. Until more specimens can be recovered we defer identifying tooth position for these specimens.

Order LIPOTYPHLA Haeckel, 1866 [32].

Suborder ERINACEOMORPHA Gregory, 1910 [33].

Family AMPHILEMURIDAE Hill, 1953 [34].

Genus *SCENOPAGUS* McKenna and Simpson, 1959 [35].

SCENOPAGUS cf. S. EDENENSIS McGrew, 1959 [31].

Specimens: TMM 41372-118, -281.

Two isolated and worn lower molars are here referred to the genus *Scenopagus* (Figure 7C). Gunnell et al. [2] report that three species of *Scenopagus* are known from Br3 (*S. curtidens*, *S. edenensis*, and *S. priscus*), but only *S. priscus* is currently known to persist into the Uintan (Ui1a and Ui1b). Both of the Whistler Squat Quarry *Scenopagus* specimens are substantially larger than *S. priscus* and *S. curtidens*, but closely match dental dimensions reported for *S. edenensis* [36]. However, the talonid breadth of 41372-118 (an m3) slightly exceeds that of *S. edenensis* and the advanced state of wear of both specimens prohibits detailed comparisons of occlusal morphology. Nonetheless, we attribute both specimens to *Scenopagus* cf. *S. edenensis* based on their large size.

Subfamily SESPEDECTINAE Novacek, 1985 [37].

Genus *PROTERIXOIDES* Stock, 1935 [38].

PROTERIXOIDES sp. nov.

Specimens: TMM 41372-219, -308.

Two isolated lower molars from the Whistler Squat Quarry represent a new sespedectine erinaceomorph that is closely allied with *Proterixoides davisi*. *P. davisi* is an endemic species from Southern California that is first known from Ui3 and last occurs in the Duchesnean [1]. The Whistler Squat Quarry specimens are slightly smaller than *P. davisi* but are much larger than the related genus *Sespedectes*. The Whistler Squat Quarry specimens also differ from *P. davisi* in being buccolingually narrower, in possessing a mesiobuccal cingulum, in possessing a metastylid, in having a more oblique distal talonid margin, and in having a more projecting hypoconulid that is in closer proximity to the entoconid. Although the recovery of additional fossil material may favor attribution of these specimens to a new genus, we provisionally attribute both molars to *Proterixoides* sp. nov. A full description of this new taxon will be published separately.

Order ?PRIMATES Linnaeus, 1758 [39].

Suborder PLESIADAPIFORMES Simons and Tattersall, 1972 [40].

Family MICROSYOPIDAE Osborn and Wortman, 1892 [41].

Genus *MICROSYOPS* Leidy, 1872 [42].

MICROSYOPS ANNECTENS Marsh, 1872 [43].

Specimen: TMM 41466-7.

This partial maxilla (Figure 7D) was referred by West [24] to *Microsyops annectens*. The morphology and smaller size of the cheek teeth of this specimen are inconsistent with attribution to either *Craseops* or *Megadelphus*. Dental dimensions of TMM 41466-7 are larger than those reported for the Bridgerian species *Microsyops elegans* but are similar to those reported for *M. annectens* [44]. *M. annectens* is known from localities spanning Br2 through Ui1b, and the only other Uintan species of *Microsyops* (*M. kratos*) is slightly larger than *M. annectens* [44]. TMM 41466-7 differs from some specimens of *M. annectens* in having well-developed lingual cingulae on M2-M3 and an M2 that lacks a distinct cuspate hypocone, but these features appear to be variable in *M. annectens* [45]. TMM 41466-7 also lacks the rugose enamel that is variably present in *M. annectens* [44]. Nonetheless, the size and anatomy of this specimen favor continued attribution to *M. annectens*.

Order PRIMATES Linnaeus, 1758 [39].

Family OMOMYIDAE Gazin, 1958 [46].

Genus *OURAYIA* Gazin, 1958 [46].

*OURAYIA UINTENSIS* Osborn, 1895 [47].

Specimen: TMM 41372-301.

This specimen is an isolated m2 trigonid from an omomyid primate (Figure 7E). Its large size and substantial buccolingual breadth, low postvallid height, rounded mesial occlusal profile, high degree of bunodonty, well-developed mesiobuccal cingulid, and crenulated enamel are consistent with attribution to *Ourayia uintensis*. This species is also known from the Ui1a locality TMM 41443 (“Junction” [12]), which is stratigraphically below the Whistler Squat Quarry in the Devil’s Graveyard Formation (Figure 2) [2], [7]. In the Uinta Basin, *O. uintensis* has historically been associated with Ui2 localities [43] but is now known to also occur at the Ui3 locality WU-26 (K.E.T., pers. obs.). If these alpha taxonomic and biostratigraphic attributions are correct, then *Ourayia uintensis* persists over a longer time interval (Ui1a–Ui3) than has been previously recognized [2], [12].

Order RODENTIA Bowdich, 1821 [48].

Family ISCHYROMYIDAE Alston, 1876 [49].

Genus *THISBEMYS* Wood, 1959 [50].

*THISBEMYS PLICATUS* Wood, 1962 [51].

Specimens: TMM 41372-25, -56, -117, -122, -129, -130, -131, 133, -135, -237, -270, -274, -296, -402.

**Figure 8:**
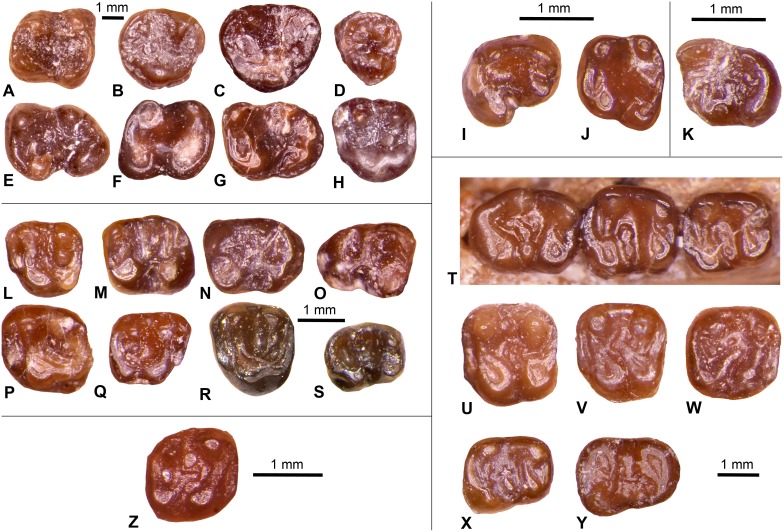
Select rodent dental specimens from the Whistler Squat local fauna. **A–H**, *Thisbemys plicatus*, (A) right m1 (TMM 41372-25), (B) right M3 (TMM 41372-56), (C) left M3 (TMM 41372-129), (D) right P4 (TMM 41372-270), (E) left m3 (TMM 41372-130), (F) right m2 (TMM 41372-131), (G) left m3 (TMM 41372-117), (H) left M2 (TMM 41372-135); **I–J**, *Microparamys minutus*, (I) right p4 (TMM 41372-298); **J**, left m1/2 (TMM 41372-260); **K**, Microparamyinae gen. et sp. indet., left m3 (TMM 41372-286); **L–S**, *Mysops boskeyi,* (L) left M1 (TMM 41372-141), (M) left m2 (TMM 41372-250), (N) left m3 (TMM 41372-253), (O) right m3 (TMM 41372-258), (P) left M2 (TMM 41372-290), (Q) right M1 (TMM 41372-307), (R) left M1 (TMM 41372-144), (S) right m1 (TMM 41372-293); **T–Y**, *Prolapsus sibilatoris*, (T) partial right mandible with m1–3, holotype (TMM 41372-179), (U) left M1 (TMM 41372-300), (V) left M1 (TMM 41372-256), (W) right M1 (TMM 41372-285), (X) right m1 (TMM 41372-291), (Y) right m3 (TMM 41372-265); **Z**, *Pauromys texensis*, right M2 (TMM 41372-279). All occlusal views.

Wood [52] identified *Thisbemys plicatus* as occurring at the Whistler Squat Quarry and two other localities that are stratigraphically lower in the Devil’s Graveyard Formation (TMM 41443 “Junction” and TMM 41444 “0.6 miles east of Junction”, Figure 2). The genus *Thisbemys* is distinctive in having cheek teeth with highly crenulated enamel [50], [51]. In fact, Korth [53] indicates that enamel crenulation is the only feature that distinguishes *Thisbemys* from *Paramys nini* and *Paramys woodi*. Wood [52] assigned the Devil’s Graveyard Formation assemblage to *Thisbemys plicatus* because the dimensions of most specimens are less than two standard deviations from the mean of the original *T. plicatus* assemblage found in the Bridger Formation of the Green River Basin, Wyoming. Whistler Squat Quarry *T. plicatus* (Figure 8A–H) have cheek tooth enamel crenulations without dentinal cores that may be obliterated by wear (typical of other *Thisbemys*) and narrow lower incisors with a faint anterior sulcus. Here we follow Wood’s [52] specific attribution because the Whistler Squat Quarry specimens resemble *T. plicatus* and differ from *T. corrugatus* in having less pronounced enamel crenulation and mandibular molars that lack a massive hypocone connected to the metaloph.

ISCHYROMYIDAE gen. et sp. nov.

Specimens: TMM 41372-259, -278, -297, -305.

Four isolated rodent teeth from the Whistler Squat Quarry represent a new genus and species of ischyromyid. Two P4s in this assemblage (41372-278 and 41372-297) were described as *Prolapsus sibilatoris* by Wood [52], but were attributed by Wilson and Runkel to “a new undescribed genus and species” ([54]: p. 2). According to J.W. Westgate (pers. comm., July, 2013), these specimens were included in an unpublished multi-authored manuscript in the early 1990s describing new Eocene rodents from Texas. In preparation for publication, these fossil rodents were cataloged using the generic name proposed in the manuscript - “*Faustimys*”. Williams and Kirk [12] subsequently listed the unpublished genus “*Faustimys*” as occurring in the Devil’s Graveyard Formation, an error that we correct here. A description of this new taxon will be published separately, and thus the Whistler Squat Quarry specimens are included here as “Ischyromyidae gen. et sp. nov.”.

Subfamily MICROPARAMYINAE Wood, 1962 [51].

Genus *MICROPARAMYS* Wood, 1959 [50].

*MICROPARAMYS MINUTUS* Wilson, 1937 [55].

Specimens: TMM 41372-260, -298.

An isolated lower molar (41372-260) and p4 (41372-298) from the Whistler Squat Quarry (Figure 8I–J) were identified by Wood [52] as *Microparamys minutus*. *M. minutus* was initially described as a species of *Paramys* by Wilson [55]. Wood [50] subsequently erected the new genus *Microparamys* to include Wilson’s assemblage and others that were discovered at various Wasatchian through Bridgerian localities across Wyoming. The teeth of *Microparamys* are very small, with most mandibular and maxillary teeth ranging in size from less than 1 mm to 2 mm in length [51]. The assemblage from the Whistler Squat Quarry is no different in this respect, with all specimens less than 2.0 mm in mesiodistal length. Furthermore, these specimens exhibit occlusal morphology typical of *M. minutus*. In particular, 41372-260 resembles other lower molars of *M. minutus* in exhibing a wide talonid basin that is defined mesially by an anterolophid, prominent protoconid, and metaconid and distally by a conspicuous entoconid, a distinct posterolophid, and prominent hypoconid.

MICROPARAMYINAE gen. et sp. indet.

Specimen: TMM 41372-286.

This single specimen of a left lower third molar (Figure 8K) was referred to “*Lophiparamys* sp. indet.” by Wood [52]. *Lophiparamys* has occlusal anatomy that is similar to *Microparamys* but is diagnosed as a separate genus primarily on the basis of strong enamel crenulation [51]. Wood [52] argued that the high metaconid and enamel crenulation of the Whistler Squat Quarry specimen favor attribution to *Lophiparamys*. However, a high metaconid is not unusual for the genus *Microparamys*, and crenulations are sometimes found in the talonid basins of *Microparamys* specimens (K.E.T., pers. obs.). TMM 41372-286 is too worn to permit further comparisons of occlusal morphology with *Lophiparamys* and *Microparamys*, and we therefore refer this specimen to the Microparamyinae gen. et sp. indet.

Family CYLINDRODONTIDAE Miller and Gidley, 1918 [56].

Genus *MYSOPS* Leidy, 1871 [57].

*MYSOPS BOSKEYI* Wood, 1973 [52].

Specimens: TMM 41372-47, -137, -138, -139, -140, -141, -142, -144, -145, -146, -147, -148, -149, -150, -249, -250, -251, -253, -257, -258, 261, -264, -267, -268, -271, -272, -273, -275, -276, -277, -280, -282, -283, -290, -293, -303, -307, -376, -378, -408, -470, -473, -773, -777, -779, -783, -786, -787, -788, -789, -789.

*Mysops boskeyi* is currently only known from the lower member of the Devil’s Graveyard Formation [7] (Figure 2). This large assemblage of *M. boskeyi* from the Whistler Squat Quarry includes the holotype and many additional specimens that Wood [52] used to diagnose the species (Figure 8L–S). All specimens are isolated teeth. According to Korth [58], *Mysops* is the earliest occurring cylindrodontid genus. Wood [52] describes *M. boskeyi* as a high-crowned species of *Mysops*, with a clear difference in height between the trigonid and talonid that is not characteristic of later cylindrodontids. By contrast. Korth [58] suggested that *M. boskeyi* should be referred to *Pareumys*, which would make “*Pareumys boskeyi*” the most primitive member of this genus. Although we acknowledge Korth’s [58] opinion here, in the absence of a stronger formal argument for transferring the species to *Pareumys* we have chosen to retain *M. boskeyi* from the Whistler Squat Quarry in the genus *Mysops*.

Family SCIURAVIDAE Miller and Gidley, 1918 [56].

Genus *PROLAPSUS* Wood, 1973 [52].

PROLAPSUS SIBILATORIS Wood, 1973 [52].

Specimens: TMM 41372-179, -252, -256, -262, -263, -265, -266, -269, -284, -285, -291, -295, -299, -300, -304, -381, -778, -782.

Wood [52] originally named *Prolapsus sibilatoris* based on specimens recovered exclusively from the Whistler Squat Quarry (Figure 8T–Y). However, Wilson and Runkel [54] described additional specimens of *P. sibilatoris* from localities in the Devil’s Graveyard and Canoe Formations that span the entire Uintan (Ui1–Ui3). The only other described member of the genus (*P. junctionis*) has a similar geographic and temporal distribution [54], and thus known occurrences of *Prolapsus* are currently restricted to the Big Bend region of Texas. Wood [52] considered *Prolapsus* to be an early ancestor of extant caviomorphs, but later studies have shown that *Prolapsus* is a sciuravid and a probable sister taxon to *Knightomys*, a widely distributed genus known from the Wasatchian and Bridgerian [54], [58]. While Wood [52] did not identify a family for this taxon, the sciuravid affinities of *P. sibilatoris* are clearly indicated by (1) its quadritubercular upper molars and (2) its quadrangular lower molars with three transverse crests derived from cingulids and transversely expanded cusps.

Genus *PAUROMYS* Troxell, 1923 [59].

*PAUROMYS TEXENSIS* Walton, 1993 [60].

Specimen: TMM 41372-279.

This isolated M2 (Figure 8Z) was initially designated *Prolapsus* sp. indet. by Wood [52], although he noted that it is “much smaller” than other species of *Prolapsus*. However, TMM 41372-279 exhibits the highly lophate cusps that are typical of the genus *Pauromys*. This specimen was included by Walton [60] in the hypodigm of *Pauromys texensis*, and we see no reason to revise this attribution. All other known specimens of *P. texensis* occur at a single locality that is located higher in the Devil’s Graveyard Formation stratigraphic section (TMM 41745; “Serendipity”, Figure 2) and considered to be Ui3 in age by Robinson et al. [1].

Order CONDYLARTHRA Cope, 1881 [61].

Family HYOPSODONTIDAE Trouessart, 1879 [22].

Genus *HYOPSODUS* Leidy, 1870 [62].

HYOPSODUS sp.

Specimen: TMM 41372-227.

**Figure 9:**
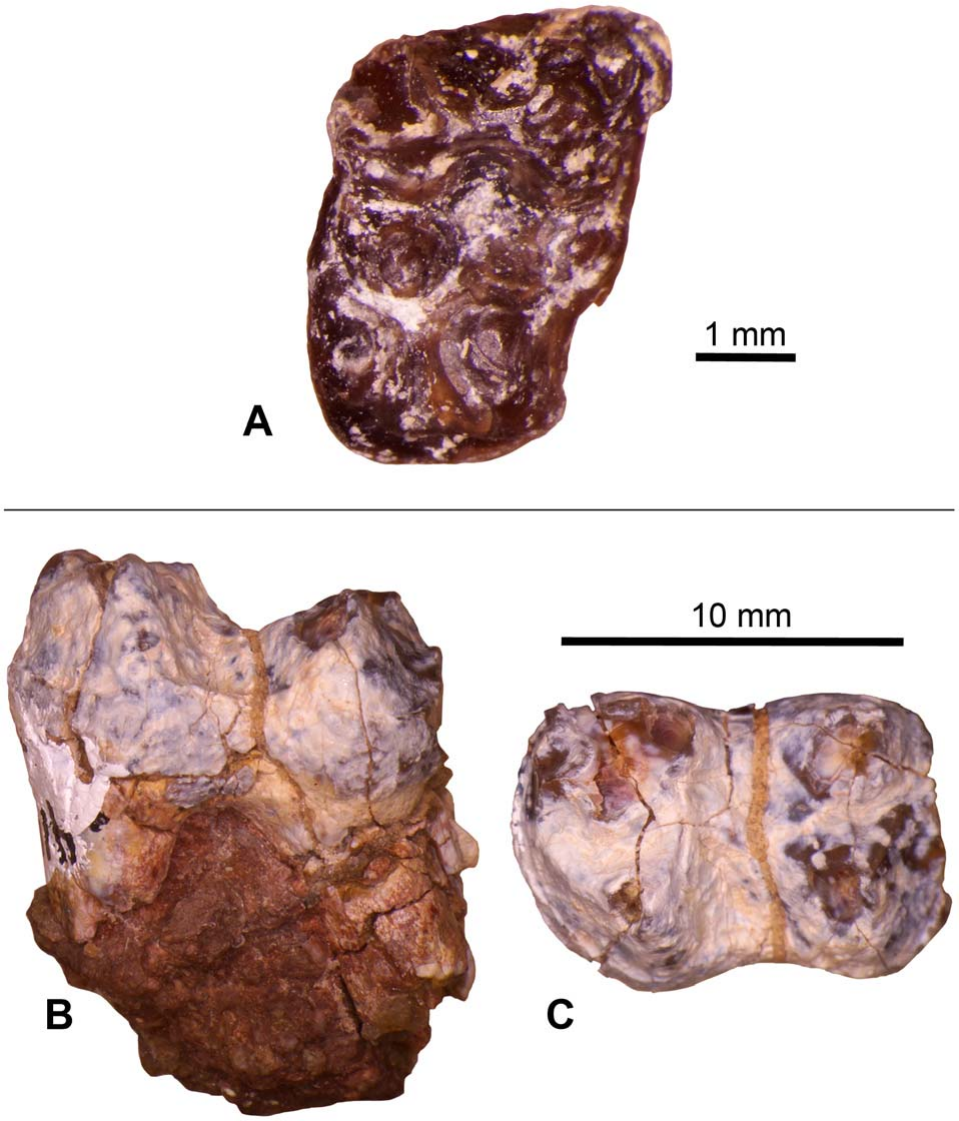
Select dental specimens from the Whistler Squat local fauna. **A**, *Hyopsodus* sp., right M2 (TMM 41372-227), occlusal view; **B–C**, *Helohyus* sp., left m1/2 (TMM 41446-12), buccal view (B) and occlusal view (C).

This isolated and worn M2 (Figure 9A) was originally attributed by West [24] to *Hyopsodus uintensis*. However, *H. uintensis* is currently only known from Ui2 and Ui3 localities [2]. The length and width of TMM 41372-227 are also below the range of all upper molar dimensions provided by Krishtalka [63] for *H. uintensis*. Given the small size of this isolated tooth and the lack of preserved anatomical detail, we have chosen to attribute this specimen to *Hyopsodus* sp.

Order ARTIODACTYLA Owen, 1848 [64].

Family HOMACODONTIDAE Marsh, 1894 [65].

HOMACODONTIDAE sp. nov.

Specimens: TMM 41372-28, -233, -234, -245, -471.

These specimens represent a new species of bunodont homacodontid artiodactyl that is most similar to the Ui3 Devil’s Graveyard Formation endemic species *Texodon meridianus* [24]. An m1 or m2 of this taxon (TMM 41372-245) was described as “*Microsus* cf. *cuspidatus*” by West [24]. This specimen resembles both *Microsus* and *Homacodon* in lacking a paraconid. However, TMM 41372-245 differs from *M. cuspidatus* and resembles “*Microsus* sp.” of Stucky [66] in possessing a complete hypolophid connecting the hypoconid and entoconid and a strongly developed cristid connecting the hypoconulid and hypolophid. In these respects, TMM 41372-245 differs from *Texodon* and the Bridgerian genus *Homacodon* but is more similar to the Bridgerian–early Uintan genus *Antiacodon* and the Ui3 genus *Auxontodon*. TMM 41372-245 also lacks the small stylid twinned with the hypoconulid seen in m2–3 of the genotype of *Microsus* (USNM 1178). An M1 or M2 of the new taxon from the Whistler Squat Quarry (TMM 41372-233) occludes well with TMM 41372-245 and is distinctive in possessing a complete postprotocone crista between the protocone and metaconule. In this respect, TMM 41372-233 resembles *Texodon* but differs from *Microsus*, *Homacodon*, *Auxontodon*, and *Antiacodon*. These two molars from the Whistler Squat Quarry are closely matched in size and morphology by a well-preserved m3 (TMM 41372-471) and two worn M3s (TMM 41372-28 and TMM 41372-234). A complete diagnosis and description of the new Whistler Squat taxon will be published separately, and we have therefore grouped the specimens here as Homacodontidae sp. nov.

Family HELOHYIDAE Marsh, 1877 [67].

Genus *HELOHYUS* Marsh, 1872 [43].

HELOHYUS sp.

Specimen: TMM 41446-12.

This m1 or m2 of a bunodont artiodactyl (Figure 9B–C) was attributed by West [24] to *Lophiohyus* based on perceived similarities to the type specimen of *Lophiohyus alticeps* [68]. According to Stucky [66], however, *L. alticeps* is a junior synonym of *Helohyus milleri*. The occlusal anatomy and precise taxonomic affinities of TMM 41446-12 are difficult to assess due to damage, particularly to the lingual portion of the trigonid and the distal portion of the talonid. Contrary to West [24], it is not possible to discern the presence and/or size of a paraconid and hypoconulid on this specimen. Nonetheless, the size and morphology of this specimen is most consistent with attribution to *Helohyus*. TMM 41446-12 is considerably larger than any known leptochoerine, antiacodontine, or homacodontine but has a length (approx. 12.5 mm) within the range of m2 lengths reported for *Helohyus* (8.4–14.3 mm) by Stucky [66]. *Helohyus* is also known from other localities spanning the early Bridgerian through early Uintan, including Ui1a and Ui1b [2], [66]. Accordingly, we attribute this specimen to *Helohyus* sp.

Family PROTOCERATIDAE Marsh, 1891 [69].

Genus *LEPTOREODON* Wortman, 1898 [70].

*LEPTOREODON MARSHI* Wortman, 1898 [70].

Specimens: TMM 41372-2, -5, -7, -10, -13, -23, -43, -44, -468, -62, -124, -125, -170, -175, -176, -177, -178, -210, -212, -214, -220, -230, -238, -242, -247, -312, -316, -361, -368, -371, -391, -395, -400, -412, -417, -434, -474, -476, -477, -486, -487, -488, -489, -490, -491, -494, -510, -519, -532, -569, -575, -581, -589, -609, -615, -616, -631, -653, -656, -658, -659, -663, -665, -666, -669, -670, -672, -676, -678, -682.

**Figure 10:**
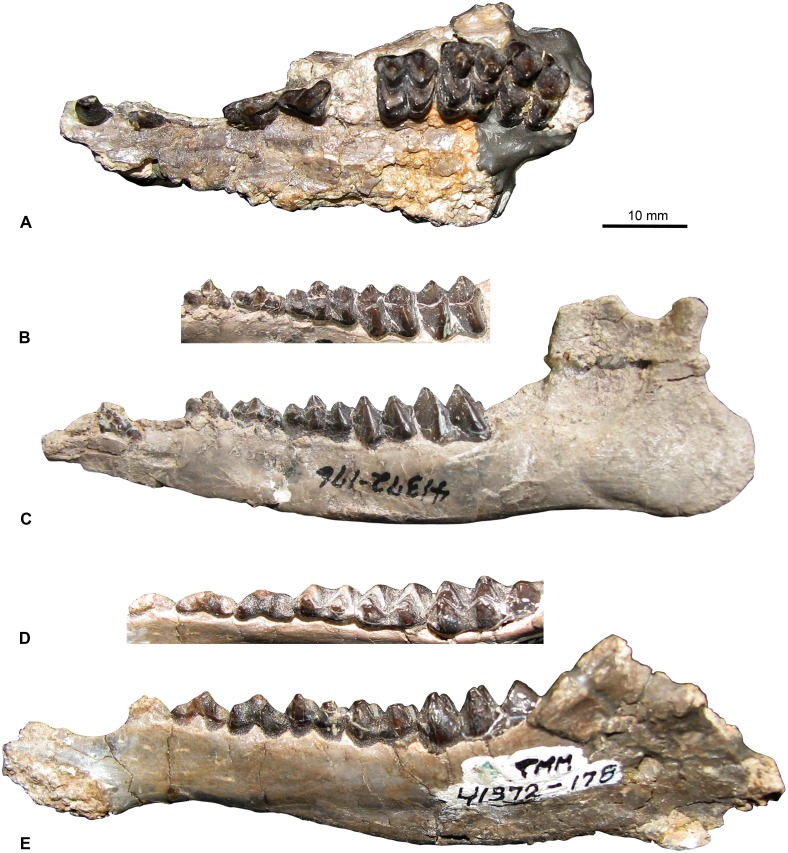
Select *Leptoreodon marshi* specimens from the Whistler Squat local fauna. **A**, left maxilla with C–P3, M1–3 (TMM 41372-175), occlusal view; **B**, left mandible with dp2–dp4, m1–m2 (TMM 41372-176), oblique occlusal view and **C**, buccal view; **D**, right mandible with p2–m3 (TMM 41372-178), oblique occlusal view and **E**, lingual view.

The presence of p4 metaconids in this large sample of *Leptoreodon marshi* from the Whistler Squat Quarry (Figure 10) help to distinguish this taxon from *Leptotragulus*, a common and slightly smaller Uintan protoceratid [71]. Wilson [72] attributed the entire Whistler Squat Quarry assemblage of *Leptoreodon* to *L. marshi* based on metric comparisons with the smaller California species *L. edwardsi* [71], [73]. We find no reason to question Wilson’s [72] assessment and we therefore retain his species attribution for these specimens.

LEPTOREODON MAJOR Golz, 1976 [73].

Specimen: TMM 41466-2.

**Figure 11:**
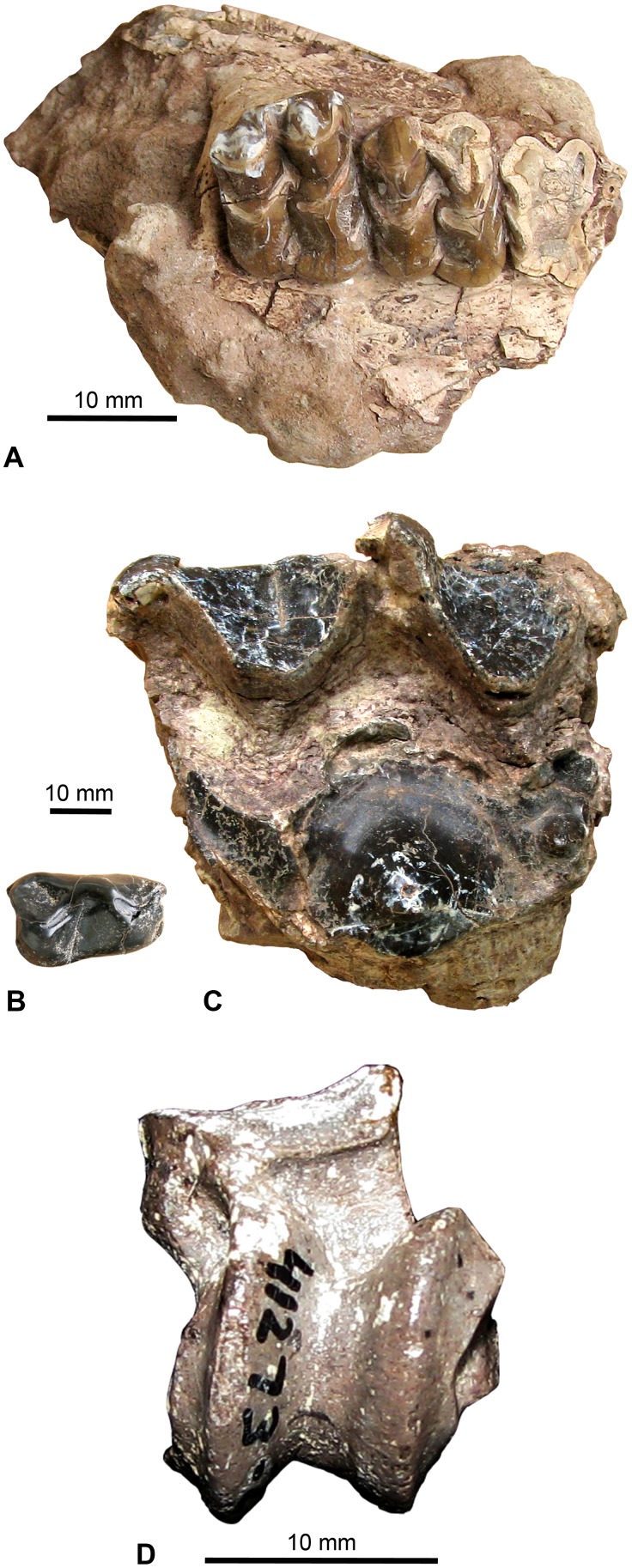
Select specimens from the Whistler Squat local fauna. **A**, *Leptoreodon major*, partial right maxilla with M1 crown base and M2-M3 (TMM 41466-2), occlusal view; **B–C**, *Protitanotherium emarginatum*, (B) right p2 (TMM 41372-536), occlusal view, (C) left M2 (TMM 41466-6), occlusal view; **D**, Equidae gen. et sp. indet., right astragalus (TMM 41372-204), superior view.

TMM 41466-2 is a partial right maxilla with M1–M3 (Figure 11A). The M1 crown is largely missing, the M2 paracone is damaged, and the M3 crown is intact. Golz [73] identified several characters distinguishing *L. major* from other species of *Leptoreodon* that are evident in this specimen, including its larger overall size and upper molars with strong cingulae, broad styles, and square occlusal profiles. We therefore attribute TMM 41466-2 to *Leptoreodon major*. This specimen is particularly important for biochronological correlation because *L. major* is an index taxon of biochron Ui1b [2].

Order PERISSODACTYLA Owen, 1848 [64].

Family BRONTOTHERIIDAE Marsh, 1873 [74].

Genus *PROTITANOTHERIUM* Hatcher, 1895 [75].

PROTITANOTHERIUM EMARGINATUM Hatcher, 1895 [75].

Specimens: TMM 41372-3, -431, -536, TMM 41466-6, -10.

This assemblage includes a large M2 (TMM 41466-6) that Wilson [76] listed in the hypodigm of *Sthenodectes australis* and three isolated lower premolars (Figure 11B–C). Mihlbachler [77] noted that although *Sthenodectes* was described by Osborn [78] as having a horn, the genotype in fact lacks horns. The holotype of *S. australis* (TMM 41723-3) [76] exhibits small elliptical frontonasal horns and is therefore more appropriately assigned to *Protitanotherium emarginatum* [77]. The anatomy and dimensions of the four isolated teeth from Whistler Squat are also consistent with attribution of these specimens to *P. emarginatum*.

Family EQUIDAE Gray, 1821 [79].

Equidae gen. et sp. indet.

Specimen: TMM 41372-204.

This isolated astragalus is clearly equid in morphology, displaying a medially projecting astragalar head, a smooth navicular facet, and a trochleated proximal articular surface that is oriented oblique to the long axis of the bone (Figure 11D). There are two genera of equids that occur during the Uintan: the early Uintan *Orohippus*, a holdover taxon from the Bridgerian, and *Epihippus*, which ranges through the Uintan [2]. In comparison with an astragalus of *Epihippus* associated with dental material from the Uinta Formation of Utah, the Whistler Squat Quarry specimen has a more elongated astragalar head (K.E.T., pers. obs.). Because no dental remains are associated with the Whistler Squat Quarry astragalus, there is some possibility that this specimen could be attributed to *Orohippus*, particularly since this taxon’s highest range datum is known from Ui1b in Wyoming [2]. Given this uncertainty whether the Whistler Squat Quarry astragalus represents *Orohippus* or *Epihippus*, we refer the specimen to Equidae gen. et sp. indet.

Family AMYNODONTIDAE Scott and Osborn, 1883 [80].

Genus *AMYNODON* Marsh, 1877 [81].

*AMYNODON ADVENUS* Marsh, 1875 [82].

Specimens: TMM 41372-2, -3, -5, -6, -8, -11, -12, -13, -14, -18, -20, -24, -45, -46, -48, -49, -50, -51, -52, -60, -61, -64, -65, -66, -68, -69, -71, -72, -73, -74, -75, -76, -77, -78, -79, -80, -81, -82, -83, -84, -85, -86, -87, -88, -89, -90, -91, -92, -94, -95, -96, -99, -100, -101, -102, -115, -127, -159, -164, -166, -171, -186, -194, -203, -206, -207, -240, -310, -325, -328, -329, -330, -331, -333, -334, -335, -336, -337, -338, -340, -342, -344, -345, -346, -347, -348, -349, -350, -351, -352, -353, -354, -355, -357, -358, -359, -360, -372, -393, -394, -399, -410, -413, -414, -415, -416, -478, -421, -422, -426, -428, -429, -430, -437, -438, -439, -440, -441-, -442, -443, -444, -446, -451, -452, -453, -454, -455, -456, -457, -458, -459, -460, -461-, 462, -492, -493, -497, -498, -500, -501, -504, -505, -507, -508-, -509, -511, -512, -514, -520, -524, -526, -533, -535, -537, -538, -539, -540, -542, -544, -545, -547, -548, -549, -550, -551, -552, -553, -554, -555, -556, -557, -558, -561, -566, -567, -570, -571, -572, -577, -578, -579, -582, -583, -585, -586, -591, -594, -596, -598, -599, -600, -601, -602, -603, -604, -605, -606, -610, -611, -612, -613, -617, -618, -619, -621, -622, -623, -624, -624, -626, -627, -628, -629, -630-, 637, -639, -644, -645, -646, -649, -650, -651, -652, -677, -684, -684, -685, -686, -687, -689, -690, -691, -692, -693, -694, -695, -696, -697, -698, -699, -700, -701, -702, -703, -704, -705, -706, -7070, -708, -709, -710, -711, -712, -713, -714, -716, -717, -718, -719, -720, -721, -722, -723, -724, -725, -726, -740, -742, -744, -745, -746, -747, -748, -750, -753, -755, -756, -757, -758, -759, -760, -761, -762, -763, -764, -770, 771, -772, -790, -791, -793, -795, -796, -797, -798, -800, -802, -803, -804, -806, -809, -810, -814.

**Figure 12:**
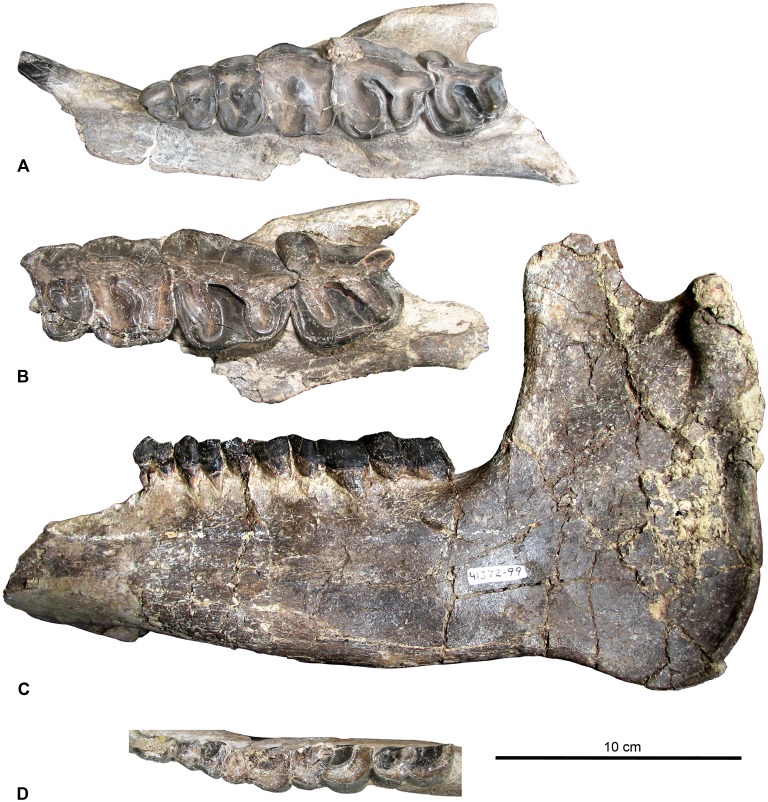
Select *Amynodon advenus* specimens from the Whistler Squat local fauna. **A**, partial left maxilla with C–M3 (TMM 41372-45), occlusal view; **B**, partial left maxilla with P4–M3 (TMM 41372-72), occlusal view; **C**, partial left mandible with p3–m3 (TMM 41372-99), buccal view and **D**, occlusal view.

This large assemblage from the Whistler Squat Quarry includes multiple skull fragments (Figure 12) from at least 11 individuals, multiple isolated teeth from all dental loci, and a large sample of postcrania. Many of these specimens were identified as *Amynodon advenus* by Wilson and Schiebout [83] in their description of the amynodontids of Trans-Pecos Texas. *A. advenus* is an index species of biochron Ui1b and is known from numerous Ui1b–Ui3 localities throughout North America [2]. The Whistler Squat Quarry cranial material shows features typical of the genus *Amynodon*, including a large preorbital fossa, a long preorbital region of the skull, and a nasal incision terminating at the level of the diastema [84]. Two species of *Amynodon* are currently recognized from the Uintan [83], [84]: *A. advenus* and *A. reedi*. The main feature that distinguishes these two species is size, with *A. reedi* approximately 25% smaller than *A. advenus* [83]. Because the Whistler Squat Quarry *Amynodon* assemblage falls within the size range of *A. advenus*, we see no reason the revise the specific attribution of Wilson and Schiebout [83].

Order CREODONTA Cope, 1875 [85].

Family HYAENODONTIDAE Leidy, 1869 [86].

Genus *SINOPA* Leidy, 1871 [57].

*SINOPA MAJOR* Leidy, 1871 [57].

Specimen: TMM 41466-9.

**Figure 13:**
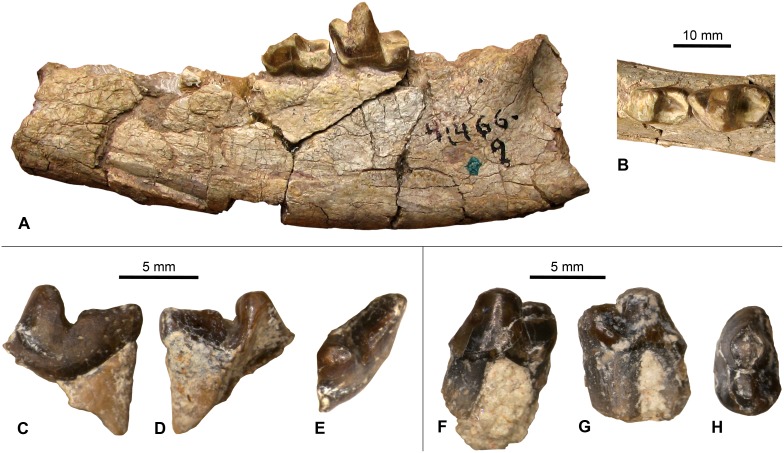
Select dental specimens from the Whistler Squat local fauna. **A-B**, *Sinopa major*, partial left mandible with m1–m2 (TMM 41466-9), buccal view (A) and occlusal view (B); **C–E**, Miacidae gen. et sp. indet., partial left P4 (TMM 41372-389), lingual view (C), buccal view (D), and occlusal view (E); **F–H**, *Miocyon* sp., left p4 (TMM 41372-367), buccal view (F), lingual view (G), and occlusal view (H).

This partial mandibular ramus with a broken m1 and worn m2 (Figure 13A–B) was referred to “?*Proviverra major*” by Gustafson [87]. The same specimen was attributed to *Sinopa major* by Gunnell [88]. Gustafson [87] published dental metrics showing that TMM 41466-9 is similar in size to Bridgerian specimens of *Sinopa major* (including the holotype YPM 11878). Our review of more detailed descriptions of this species [89] indicate that TMM 41466-9 is comparable in morphology to specimens originally referred to *Sinopa major* [88], [90]. Recent taxonomic revisions have indicated that *Proviverra* is a genus restricted to Europe, and we therefore retain Gunnell’s [88] attribution of this specimen to *Sinopa major* [88], [90].

Order CARNIVORAMORPHA Wyss and Flynn, 1993 [91].

Family MIACIDAE Cope, 1880 [92].

MIACIDAE gen. et sp. indet.

Specimen: TMM 41372–389.

This specimen is the distobuccal portion of a carnivoramorph P4, including the paracone and metastylar blade (Figure 13C–E). Although the mesial portion of the crown is missing, the slightly conical morphology of the paracone and the rounded, more open carnassial notch, not typical of taxa known in Viverravidae, favors attribution of this tooth to the Miacidae [93]. We have therefore referred this specimen to Miacidae gen. et sp. indet.

Genus *MIOCYON* Matthew, 1909 [94].

MIOCYON sp.

Specimens: TMM 41372-367.

This isolated p4 (Figure 13F–H) was tentatively referred to *Uintacyon scotti* by Gustafson [87]. However, as explained by Friscia and Rasmussen [95], the correct generic attribution for this miacid species is *Miocyon*. In the Uinta Formation of Utah, *Miocyon* is probably represented by two species that co-occur at some fossil localities: *M. vallisrubrae* and the larger species *M. scotti* [95]. Other than size, the main characters that potentially distinguish *M. vallisrubrae and M. scotti* are found on the m2, which is unknown from the Whistler Squat Quarry and equivalent localities. However, Friscia and Rasmussen [95] could not exclude the possibility that specimens of *M. vallisrubrae and M. scotti* represent a single sexually dimorphic species. Wear of the Whistler Squat Quarry specimen also hinders comparisons with known species of *Miocyon*, and we therefore refer this specimen to *Miocyon* sp.

## Discussion

In his synthesis of Eocene vertebrate faunas from Trans-Pecos Texas, Wilson [7] variously referred to the localities comprising the Whistler Squat local fauna as both “early Uintan” and as belonging to the “Uinta B” land mammal age. However, as noted by Prothero [96], Walsh [97], and Townsend et al. [98], “Uinta B” refers specifically to a local lithostratigraphic unit in the Uinta Basin that has yielded characteristically Ui2 faunas. Further confusion is introduced by Figure 3 in Wilson [7], which identifies the stratigraphically lowest localities in the Whistler Squat local fauna (e.g., “Junction”, “0.6 miles east of Junction”, and “Hen Egg Mountain”) as potentially late Bridgerian. Robinson et al. [1] also called attention to faunal differences between the “Junction localities” ( = “basal Tertiary conglomerate” localities of [1]) and the “Whistler Squat Quarry” assemblage, which these authors identified as “Ui1” and “Ui1–Ui2” respectively. Gunnell et al. [2] subsequently subdivided Ui1 into biochrons Ui1a and Ui1b, recognizing Ui1b by the first appearance of the selenodont artiodactyls *Protoreodon*, *Leptoreodon*, and *Protylopus*, the rhinocerotoid *Amynodon*, and the uintathere *Eobasileus*. Gunnell et al. ([2]: p. 313) designated the “basal Tertiary conglomerate” localities of the Devil’s Graveyard Formation as a Ui1a reference section. However, Gunnell et al. ([2]: p. 314) also designated the “Whistler Squat Local Fauna… (Wilson, 1986)” as a Ui1b reference section despite the fact that the “basal Tertiary conglomerate” localities comprise part of Wilson’s [7] Whistler Squat local fauna. Because the “basal Tertiary conglomerate” localities lack the genera identified by Gunnell et al. [2] as definitive of Ui1b (cf. [7]: table 1), Gunnell et al. [2] intended to include only the Whistler Squat Quarry and equivalent localities (cf. [7]: table 2) as a Ui1b reference section (P.C. Murphey, pers. comm., March, 2014) in accord with Walton’s alternative use of the “Whistler Squat local fauna” [5], [6].

**Figure 14:**
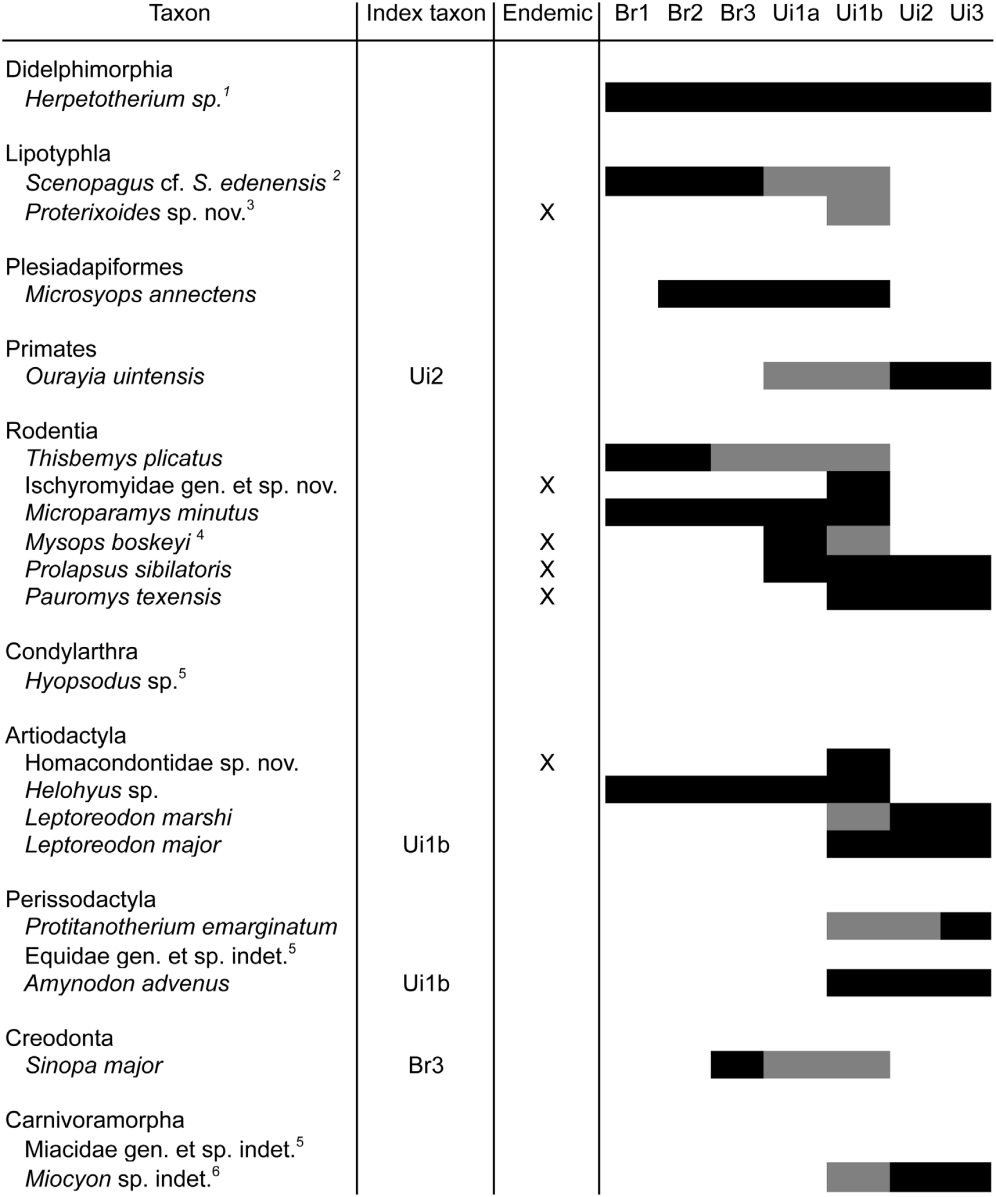
Whistler Squat faunal list and associated Bridgerian and Uintan biochronological zone ranges. Mammalian taxa identified from Whistler Squat local fauna localities TMM-41372 and TMM-41466 and their Bridgerian (Br) and Uintan (Ui) biochronological zone ranges based on Wilson [7], Wilson and Runkel [51], Walton [60], Williams and Kirk [12], Robinson [1], Gunnell et al. [2], and this study. Gray bars indicate range extension of non-endemic taxa compared to Robinson et al. [1] and Gunnell et al. [2]. ^1^ *Herpetotherium* species candidates range throughout the Bridgerian and Uintan, ^2^Range listed is for *S. edenensis*, ^3^Endemic species, but extends LRD of genus from Ui3. ^4^Endemic species, but extends HRD of genus from Ui1a. ^5^Range is genus/species specific. ^6^Extends LRD of genus from Ui2.

Our revised faunal list for TMM 41372 and TMM 41466 (Figure 14) reinforces the conclusion that the fauna from Whistler Squat Quarry and equivalent localities in the Devil’s Graveyard Formation are attributable to biochron Ui1b. Most significantly, our revised faunal list includes two index species of biochron Ui1b: *Leptoreodon major* and *Amynodon advenus* [2]. As noted previously, the first appearances of the genera *Leptoreodon* and *Amynodon* help to define Ui1b as distinct from Ui1a. Our revised faunal list also includes one genus (*Microsyops*) and one species (*Microparamys minutus*) that are last known to occur in Ui1b [2].

As discussed by Gunnell et al. [2], other Ui1b localities in southern California and the northern Rocky Mountains demonstrate striking patterns of regional endemism. The mammalian fauna from the Whistler Squat Quarry is similar in this respect, with two new undescribed taxa (a homacodontid artiodactyl related to *Texodon meridianus* and a sespedectine erinaceomorph related to *Proterixoides davisi*) currently known only from the Whistler Squat Quarry (TMM 41372). Similarly, the sciuravid rodent species *Pauromys texensis* and the cylindrodontid rodent species *Mysops boskeyi* are currently only known from the Whistler Squat Quarry and other localities in the Devil’s Graveyard Formation [52], [60]. Two additional genera of rodents (a new ischyromyid genus catalogued previously under the invalid genus name “*Faustimys*” and the sciuravid *Prolapsus*) are currently only known from the Whistler Squat Quarry and other middle Eocene localities in Texas. Research in progress describing the anatomy and phylogenetic relationships of the various new regionally endemic taxa from the Whistler Squat Quarry will help to further clarify the pattern of faunal endemism characteristic of the early Uintan in Texas.

This reanalysis of Whistler Squat Quarry specimens has also led to the extension of biochronological zone ranges to Ui1b for several taxa compared to recent compilations by Robinson et al. [1] and Gunnel et al. [2] (Figure 14). The highest range datum (HRD) is extended for four taxa. If *Scenopagus edenensis* is represented at the Whistler Squat Quarry, then the HRD for this species and *Sinopa major* are here extended from Br3 to Ui1b. Wilson’s [7] previous documentation of both *Thisbemys plicatus* and *Mysops boskeyi* at the Whistler Squat Quarry extends the HRD of *T. plicatus* and the genus *Mysops* to Ui1b from Br2 and Ui1a, respectively [2]. The lowest range datum (LRD) is also extended for five taxa. Wilson [7] documented *Leptoreodon marshi* at the Whistler Squat Quarry, and Williams and Kirk [12] previously documented *Ourayia uintensis* from the Ui1a “Junction” locality, extending the LRD for both species from Ui2 [2]. The reallocation of *Sthenodectes australis* specimens [7] to *Protitanotherium emarginatum* [77] extends the LRD for the genus from Ui3 and the presence of *Proterixoides* and *Miocyon* at the Whistler Squat Quarry extends the LRD for these genera to Ui1b from Ui3 and Ui2, respectively [1], [2].

Well-characterized faunal assemblages in a highly resolved chronostratigraphic framework are key to understanding the Bridgerian to Uintan faunal transition. Our study provides an essential first step in this process by presenting a well-constrained chronostratigraphy for an early Uintan locality. New geochronological analysis of tephra bracketing the Whistler Squat Quarry assemblage has provided a high-precision age estimate of 44.88±0.04 Ma, a significant improvement compared to the previous age range that spanned almost 5 million years (43.2–47.9 Ma with ±1σ uncertainty). These new dates are compatible with magnetically reversed sediments at the site [5], [6] attributable to C20r (43.505–45.942 Ma [99]). The revised date for the Whistler Squat Quarry is also consistent with a date of 46.80±0.08 Ma for a stratigraphically lower basalt outcropping on the southeast side of Hen Egg Mountain [100] (recalibrated to Fish Canyon standard of 28.201±0.046 Ma) that is likely contemporaneous with the Alamo Creek Basalt of the Chisos Formation and Basalt A of the Canoe Formation [9], [10], [100], [101]. Significantly, this basalt overlies the Hen Egg Mountain fossil localities (TMM 42028 and TMM 42287) that comprise part of the Basal Tertiary local fauna [5], [7]. If Wilson [7] and Runkel [9] are correct that the Hen Egg Mountain fossil localities are roughly contemporaneous with TMM 41443 (“Junction”) and TMM 41444 (“0.6 miles east of Junction”), then the Ui1b localities of the Whistler Squat local fauna are ∼2 Ma younger than the localities of the Basal Tertiary local fauna.

Renewed fieldwork in late Uintan (Ui3) deposits of the DGF has yielded abundant fossil remains, including several new primate species [12], [102]. New ^40^Ar/^39^Ar and paleomagnetic analyses of these Ui3 sediments are currently in progress. The work presented here provides the first ^40^Ar/^39^Ar date directly associated with a fossil assemblage attributed to the Ui1b biochron and the most precise radiometric date directly associated with an early Uintan assemblage. This study also highlights the need for thorough reexaminations of collections with decades-old taxonomic identifications in order to properly document patterns of endemism and biochrononological ranges in the Eocene. As noted by Prothero [96], the DGF and correlative deposits in the Trans-Pecos region hold the potential to significantly increase our knowledge of Uintan biochronology and biogeography. With the inclusion of the localities of the Basal Tertiary local fauna, the Devil’s Graveyard Formation is an ideal setting to document the Bridgerian–Uintan transition (biochrons Br3-Ui1b) within a well-dated continuous sequence. This objective is the focus of ongoing and future work. Combined with a refined understanding of the Bridger Formation of Wyoming (e.g., [4], [103]), continued paleontological and geochronological research in the DGF may also significantly improve our understanding of the details of the Bridgerian–Uintan transition across North America, including any geographical variation in the timing of this transition.

## Supporting Information

Table S1 **Complete ^40^Ar/^39^Ar analytical data for samples JW-1 and JW-2.**
(XLSX)

## References

[1] Robinson P, Gunnell GF, Walsh SL, Clyde WC, Storer JE et al.. (2004) Wasatchian through Duchesnean biochronology. In: Woodburne MO, editor. Late Cretaceous and Cenozoic Mammals of North America: Biostratigraphy and Geochronology. Berkeley: University of California Press. 106–155.

[2] Gunnell GF, Murphey PC, Stucky RK, Townsend KEB, Robinson P, et al (2009) Biostratigraphy and biochronology of the latest Wasatchian, Bridgerian, and Uintan North American Land Mammal “Ages”. In: Museum of Northern Arizona Bulletin Albright LB, editor. Papers on Geology, Vertebrate Paleontology, and Biostratigraphy in Honor of Michael O Woodburne. 65: 279–330.

[3] Townsend KEB, Gunnell GF (2009) Regional trends in mammalian paleoecology during the Uintan (middle Eocene) North American Land Mammal Age. Cincinnati Museum Center Scientific Contributions, 9th North American Paleontological Convention 3: 62.

[4] Townsend KEB, Rasmussen DT, Murphey PC, Evanoff E (2010) Middle Eocene habitat shifts in the North American western interior: a case study. Palaeogeography, Palaeoclimatology, Palaeoecology 297: 144–158.

[5] Walton AH (1986) Magnetostratigraphy and the Ages of Bridgerian and Uintan Faunas in the Lower and Middle Members of the Devil’s Graveyard Formation, Trans-Pecos Texas (M.A. thesis). Austin: University of Texas at Austin. 134 p.

[6] Walton AH (1992) Magnetostratigraphy and geochronology of the lower and middle members of the Devil’s Graveyard Formation (middle Eocene), Trans-Pecos Texas. In: Prothero DR, Berggren WA, editors. Eocene-Oligocene Climatic and Biotic Evolution. Princeton: Princeton University Press. 74–87.

[7] Wilson JA (1986) Stratigraphic occurrence and correlation of early Tertiary vertebrate faunas, Trans-Pecos Texas: Agua Fria-Green Valley areas. Journal of Vertebrate Paleontology 6: 350–373.

[8] Stevens JB, Stevens MB, Wilson JA (1984) Devil’s Graveyard Formation (new), Eocene and Oligocene age, Trans-Pecos Texas. Texas Memorial Museum Bulletin 32: 1–21.

[9] Runkel AC (1990) Stratigraphy and depositional history of Late Cretaceous and Paleogene rocks, Trans-Pecos Texas. In: Dickerson PW, Stevens MS, Stevens JB, editors. Geology of the Big Bend and Trans-Pecos Region South Texas Geological Society Fieldtrip Guidebook: 117–146.

[10] Runkel AC (1990) Lateral and temporal changes in volcanogenic sedimentation; analysis of two Eocene sedimentary aprons, Big Bend region, Texas. Journal of Sedimentary Petrology 60: 747–760.

[11] Henry CD, Davis L, L., Kunk MJ, McIntosh WC (1998) Tertiary volcanism of the Bofecillos Mountains and Big Bend Ranch State Park, Texas: revised stratigraphy and ^40^Ar/^39^Ar geochronology. The University of Texas at Austin, Bureau of Economic Geology, Report of Investigations 253: 1–74.

[12] Williams BA, Kirk EC (2008) New Uintan primates from Texas and their implications for North American patterns of species richness during the Eocene. Journal of Human Evolution 55: 927–941. 18835008 10.1016/j.jhevol.2008.07.007

[13] McDowell FW (1979) Potassium-argon dating in the Trans-Pecos Texas volcanic field. In: Cenozoic Geology of the Trans-Pecos Volcanic Field of Texas The University of Texas at Austin, Bureau of Economic Geology, Guidebook Walton AW, Henry CD, editors. 19: 10–18.

[14] Henry CD, McDowell FW, Price JG, Smyth RC (1986) Compilation of potassium-argon ages of Tertiary igneous rocks, Trans-Pecos Texas. The University of Texas at Austin, Bureau of Economic Geology, Geological Circular 86–2: 1–34.

[15] Kuiper KF, Deino AL, Hilgen FJ, Krijgsman W, Renne P, et al (2008) Synchronizing rock clocks of Earth history. Science 320: 500–504. 18436783 10.1126/science.1154339

[16] Best MG, Christiansen EH, Deino AL, Gromme CS, Tingey DG (1995) Correlation and emplacement of a large, zoned, discontinuously exposed ash flow sheet: The ^40^Ar/^39^Ar chronology, paleomagnetism, and petrology of the Pahranagat Formation, Nevada. Journal of Geophysical Research: Solid Earth 100: 24593–24609.

[17] Steiger RH, Jäger E (1977) Subcommission on geochronology: Convention on the use of decay constants in geo- and cosmochronology. Earth and Planetary Science Letters 36: 359–362.

[18] Deino A, Potts R (1990) Single-crystal ^40^Ar/^39^Ar dating of the Olorgesailie Formation, southern Kenya rift. Journal of Geophysical Research: Solid Earth and Planets 95: 8453–8470.

[19] Deino AL, Renne PR, Swisher CC (1998) ^40^Ar/^39^Ar dating in paleoanthropology and archaeology. Evolutionary Anthropology 6: 63–75.

[20] Deino AL, Scott G, Saylor B, Mulugeta A, Angelini JD, et al (2010) 40Ar/39Ar dating, paleomagnetism, and tephrochemistry of Pliocene strata of the hominid-bearing Woranso-Mille area, west-central Afar Rift, Ethiopia. Journal of Human Evolution 58: 111–126. 20034653 10.1016/j.jhevol.2009.11.001

[21] Gill T (1872) Arrangement of the families of mammals with analytical tables. Smithsonian Miscellaneous Collections 11: 1–98.

[22] Trouessart EL (1879) Catalogue des mammiféres vivants et fossiles. Insectivores. Revue et Magasin de Zoologie Pure et Appliquée (serie 3) 7: 219–285.

[23] Cope ED (1873) Third notice of extinct Vertebrata from the Tertiary of the plains. Paleontological Bulletin 16: 1–8.

[24] West RM (1982) Fossil mammals from the lower Buck Hill Group, Eocene of Trans-Pecos Texas: Marsupicarnivora, Primates, Taeniodonta, Condylarthra, bunodont Artiodactyla, and Dinocerata. Texas Memorial Museum, Pearce-Sellards Series 35: 1–20.

[25] Krishtalka L, Stucky RK (1983) Paleocene and Eocene marsupials of North America. Annals of Carnegie Museum 52: 229–263.

[26] Korth WW (1994) Middle Tertiary marsupials (Mammalia) from North America. Journal of Paleontology 68: 376–397.

[27] Rothecker J, Storer JE (1996) The marsupials of the Lac Pelletier lower fauna, Middle Eocene (Duchesnean) of Saskatchewan. Journal of Vertebrate Paleontology 16: 770–774.

[28] Troxell EL (1923) A new marsupial. American Journal of Science (Series 5) 5: 507–510.

[29] Storer JE (1984) Mammals of the Swift Current Creek Local Fauna (Eocene: Uintan), Saskatchewan. Natural History Contributions, Saskatchewan Museum of Natural History 7: 1–158.

[30] Rose KD, Chew AE, Dunn RH, Kraus MJ, Fricke HC, et al (2012) Earliest Eocene mammalian fauna from the Paleocene-Eocene Thermal Maximum at Sand Creek Divide, southern Bighorn Basin, Wyoming. University of Michigan Papers on Paleontology 36: 1–122.

[31] McGrew PO (1959) Leptictidae. In: Bulletin of the American Museum of Natural History 117 McGrew PO, editor. The Geology and Paleontology of the Elk Mountain and Tabernacle Butte Area, Wyoming. 117–176: 148–151.

[32] Haeckel E (1866) Systematische Einleitung in die allgemeine Entwicklungsgeschichte. Generelle morphologie der organismem. Berlin: Georg Reimer. 140 p.

[33] Gregory WK (1910) The orders of mammals. Bulletin of the American Museum of Natural History 27: 1–524.

[34] Hill WCO (1953) Primates: Comparative Anatomy and Taxonomy I - Strepsirhini. Edinburgh: Edinburgh University Press. 798 p.

[35] McKenna MC, Simpson GG (1959) A new insectivore from the middle Eocene of Tabernacle Butte, Wyoming. American Museum Novitates 1953: 1–12.

[36] Krishtalka L (1976) Early Tertiary Adapisoricidae and Erinaceidae (Mammalia, Insectivora) of North America. Bulletin of Carnegie Museum of Natural History 1: 1–40.

[37] Novacek MJ (1985) The Sespedectinae, a new subfamily of hedgehog-like insectivores. American Museum Novitates 2822: 1–24.

[38] Stock C (1935) Insectivora from the Sespe uppermost Eocene, California. Proceedings of the National Academy of Sciences 21: 214–219. 10.1073/pnas.21.4.214 PMC1076567 16587961

[39] Linnaeus C (1758) Systema Maturae per Regna Tria Naturae, Secundum Classes, Ordines, Genera, Species, cum Characteribus, Differentiis, Synonymis, Locis. Stockholm: Laurentii Salvii. 824 p.

[40] Simons EL, Tattersall I (1972) In: Simons EL, editor. Primate Evolution: An Introduction to Man’s Place in Nature. New York: Macmillan. p. 284.

[41] Osborn HF, Wortman JL (1892) Fossil mammals of the Wahsatch and Wind River beds: collection of 1891. Bulletin of the American Museum of Natural History 4: 81–147.

[42] Leidy J (1872) Remarks on fossils from Wyoming. Proceedings of the Academy of Natural Sciences of Philadelphia 24: 19–21.

[43] Marsh OC (1872) Preliminary description of new Tertiary mammals. American Journal of Science and Arts (Series 3) 4: 202–224.

[44] Gunnell GF (1989) Evolutionary history of Microsyopoidea (Mammalia, ?Primates) and the relationship between plesiadapiformes and primates. University of Michigan, Papers on Paleontology 27: 1–157.

[45] Szalay FS (1969) Mixodectidae, Microsyopidae, and the insectivore-primate transition. Bulletin of the American Museum of Natural History 140: 193–330.

[46] Gazin CL (1958) A review of the middle and upper Eocene primates of North America. Smithsonian Miscellaneous Collections 136: 1–112.

[47] Osborn HF (1895) Fossil mammals of the Uinta Basin: expedition of 1894. Bulletin of the American Museum of Natural History 7: 71–105.

[48] Bowdich TE (1821) An analysis of the natural classifications of Mammalia, for the use of students and travellers. Paris: J. Smith. 115 p.

[49] Alston ER (1876) On the classification of the Order Glires. Proceedings of the Zoological Society of London 44: 61–98.

[50] Wood AE (1959) Rodentia. In: The Geology and Paleontology of the Elk Mountain and Tabernacle Butte Area, Wyoming Bulletin of the American Museum of Natural History McGrew PO, editor. 117: 157–169.

[51] Wood AE (1962) Early Tertiary rodents of the family Paramyidae. Transactions of the American Philosophical Society 52: 1–261.

[52] Wood AE (1973) Eocene rodents, Pruett Formation, southwest Texas: Their pertinence to the origin of the South American Caviomorpha. Texas Memorial Museum, Pearce-Sellards Series 20: 1–40.

[53] Korth WW (1984) Earliest Tertiary evolution and radiation of rodents in North America. Bulletin of Carnegie Museum of Natural History 24: 1–71.

[54] Wilson JA, Runkel AC (1991) *Prolapsus*, a large sciuravid rodent and new eomyids from the late Eocene of Trans-Pecos Texas. Texas Memorial Museum, Pearce-Sellards Series 48: 1–29.

[55] Wilson RW (1937) Two new Eocene rodents from the Green River Basin, Wyoming. American Journal of Science (Series 5) 34: 447–456.

[56] Miller GS, Gidley JW (1918) Synopsis of the supergeneric groups of rodents. Journal of the Washington Academy of Sciences 8: 431–448.

[57] Leidy J (1871) Remains of extinct mammals from Wyoming. Proceedings of the Academy of Natural Sciences of Philadelphia 23: 113–116.

[58] Korth WW (1994) The Tertiary Record of Rodents in North America. New York: Plenum. 319 p.

[59] Troxell EL (1923) *Pauromys perditus*, a small rodent. American Journal of Science (Series 5) 5: 155–156.

[60] Walton AH (1993) *Pauromys* and other small Sciuravidae (Mammalia: Rodentia) from the middle Eocene of Texas. Journal of Vertebrate Paleontology 13: 243–261.

[61] Cope ED (1881) A new type of Perissodactyla. American Naturalist 15: 1017–1018.

[62] Leidy J (1870) Remarks on a collection of fossils from the western territories. Proceedings of the Academy of Natural Sciences of Philadelphia 22: 109–110.

[63] Krishtalka L (1979) Paleontology and Geology of the Badwater Creek Area, Central Wyoming. Part 18. Revision of late Eocene *Hyopsodus* . Annals of Carnegie Museum 48: 377–389.

[64] Owen R (1848) Description of teeth and portions of jaws of two extinct anthracotherioid quadrupeds (*Hyopotamus vectianus* and *Hyop. bovinus*) discovered by the Marchioness of Hastings in the Eocene deposits on the N.W. coast of the Isle of Wight: with an attempt to develop Cuvier’s idea of the classification of pachyderms by the number of their toes. Quarterly Journal of the Geological Society of London 4: 103–141.

[65] Marsh OC (1894) Description of Tertiary artiodactyles. American Journal of Science (Series 3) 48: 259–274.

[66] Stucky RK (1998) Eocene bunodont and bunoselenodont Artiodactyla (“dichobunids”). In: Janis CM, Scott KM, Jacobs LL, editors. Evolution of Tertiary Mammals of North America. Volume 1: Terrestrial Carnivores, Ungulates, and Ungulatelike Mammals. Cambridge: Cambridge University Press. 358–374.

[67] Marsh OC (1877) The introduction and succession of vertebrate life in America. American Journal of Science and Arts (Series 3) 14: 337–378.

[68] Sinclair WJ (1914) A revision of the bunodont Artiodactyla of the middle and lower Eocene of North America. Bulletin of the American Museum of Natural History 33: 267–295.

[69] Marsh OC (1891) A horned artiodactyle (*Protoceras celer*) from the Miocene. American Journal of Science (Series 3) 41: 81–82.

[70] Wortman JL (1898) The extinct Camelidae of North America and some associated forms. Bulletin of the American Museum of Natural History 10: 93–142.

[71] Gazin CL (1955) A review of the upper Eocene Artiodactyla of North America. Smithsonian Miscellaneous Collections 128: 1–35.

[72] Wilson JA (1974) Early Tertiary vertebrate faunas, Vieja Group and Buck Hill Group, Trans-Pecos Texas: Protoceratidae, Camelidae, Hypertragulidae. Texas Memorial Museum Bulletin 23: 1–34.

[73] Golz DJ (1976) Eocene Artiodactyla of southern California. Natural History Museum of Los Angeles County, Science Bulletin 26: 1–85.

[74] Marsh OC (1873) Notice of new Tertiary mammals. American Journal of Science and Arts (Series 3) 5: 407–410, 485–488.

[75] Hatcher JB (1895) On a new species of *Diplacodon*, with a discussion of the relations of that genus to *Tetmatotherium* . American Naturalist 29: 1084–1090.

[76] Wilson JA (1977) Early Tertiary vertebrate faunas Big Bend area Trans-Pecos Texas: Brontotheriidae. Texas Memorial Museum, Pearce-Sellards Series 25: 1–15.

[77] Mihlbachler MC (2008) Species taxonomy, phylogeny, and biogeography of the Brontotheriidae (Mammalia: Perissodactyla). Bulletin of the American Museum of Natural History 311: 1–475.

[78] Osborn HF (1929) Titanotheres of ancient Wyoming, Dakota, and Nebraska. United States Geological Survey Monograph 55: 1–894.

[79] Gray JE (1821) On the natural arrangement of vertebrose animals. The London Medical Repository Monthly Journal and Review 15: 296–310.

[80] Scott WB, Osborn HF (1883) On the skull of the Eocene rhinoceros, *Orthocynodon*, and the relation of this genus to other members of the group. Contributions from the E M Museum of Geology and Archaeology of Princeton College 3: 3–22.

[81] Marsh OC (1877) Notice of some new vertebrate fossils. American Journal of Science and Arts (Series 3) 14: 249–256.

[82] Marsh OC (1875) Notice of new Tertiary mammals. American Journal of Science and Arts (Series 3) 9: 239–250.

[83] Wilson JA, Schiebout JA (1981) Early Tertiary vertebrate faunas, Trans-Pecos Texas: Amynodontidae. Texas Memorial Museum, Pearce-Sellards Series 33: 1–62.

[84] Wall WP (1998) Amynodontidae. In: Janis CM, Scott KM, Jacobs LL, editors. Evolution of Tertiary Mammals of North America. Volume 1: Terrestrial Carnivores, Ungulates, and Ungulatelike Mammals. Cambridge: Cambridge University Press. 583–588.

[85] Cope ED (1875) On the supposed Carnivora of the Eocene of the Rocky Mountains. Proceedings of the Academy of Natural Sciences of Philadelphia 27: 444–449.

[86] Leidy J (1869) The extinct mammalian fauna of Dakota and Nebraska, including an account of some allied forms from other localities, together with a synopsis of the mammalian remains of North America. Journal of the Academy of Natural Sciences of Philadelphia, series 2 7: 1–472.

[87] Gustafson EP (1986) Carnivorous mammals of the late Eocene and early Oligocene of Trans-Pecos Texas. Texas Memorial Museum Bulletin 33: 1–66.

[88] Gunnell GF (1998) Creodonta. In: Janis CM, Scott KM, Jacobs LL, editors. Evolution of Tertiary Mammals of North America. Volume 1: Terrestrial Carnivores, Ungulates, and Ungulatelike Mammals. Cambridge: Cambridge University Press. 91–109.

[89] Matthew WD (1906) The osteology of *Sinopa*, a creodont mammal of the middle Eocene. Proceedings of the United States National Museum 30: 203–233.

[90] Morlo M, Gunnell GF (2003) Small Limnocyonines (Hyaeondontidae, Mammalia) from the Bridgerian, middle Eocene of Wyoming: Thinocyon, Prolimnocyon, and Iridodon, new genus. Contributions from the Museum of Paleontology, University of Michigan 31: 43–78.

[91] Wyss AR, Flynn JJ (1993) A phylogenetic analysis and definition of the Carnivora. In: Szalay FS, Novacek MJ, McKenna MC, editors. Mammal Phylogeny: Placentals. New York: Springer-Verlag. 32–52.

[92] Cope ED (1880) The bad lands of the Wind River and their fauna. American Naturalist 14: 745–748.

[93] Flynn JJ, Galiano H (1982) Phylogeny of early Tertiary Carnivora, with a description of a new species of *Protictis* from the middle Eocene of northwestern Wyoming. American Museum Novitates 2725: 1–64.

[94] Matthew WD (1909) The Carnivora and Insectivora of the Bridger Basin, middle Eocene. Memoirs of the American Museum of Natural History 9: 291–567.

[95] Friscia AR, Rassmussen DT (2010) Middle Eocene Carnivoramorpha of the Uinta Basin, Utah. Annals of Carnegie Museum 79: 51–63.

[96] Prothero DR (1996) Magnetostratigraphy of the Eocene-Oligocene transition in Trans-Pecos Texas. In: Prothero DR, Emry RJ, editors. The Terrestrial Eocene-Oligocene Transition in North America. Cambridge: Cambridge University Press. 189–198.

[97] Walsh SL (1996) Middle Eocene mammalian faunas of San Diego County, California. In: Prothero DR, Emry RJ, editors. The Terrestrial Eocene-Oligocene Transition in North America. Cambridge: Cambridge University Press. 75–119.

[98] Townsend KEB, Friscia AR, Rassmussen DT (2006) Stratigraphic distribution of upper middle Eocene fossil vertebrate localities in the eastern Uinta Basin, Utah, with comments on Uintan biostratigraphy. Mountain Geologist 43: 115–134.

[99] Ogg JG (2012) Geomagnetic Polarity Time Scale. In: Gradstein FM, Ogg JG, Schmitz MD, Ogg GM, editors. The Geological Time Scale 2012. Amsterdam: Elsevier. 85–114.

[100] Miggins DP (2009) Temporal and Geochemical Insights Related to Volcanic and Plutonic Activity Within Big Bend National Park, Texas (Ph.D. dissertation). El Paso: University of Texas at El Paso. 286 p.

[101] Henry CD, Price JG, Miser DE (1989) Geology and Tertiary igneous activity of the Hen Egg Mountain and Christmas Mountains Quadrangles, Big Bend Region, Trans-Pecos Texas. The University of Texas at Austin, Bureau of Economic Geology, Report of Investigations 183: 1–105.

[102] Kirk EC, Williams BA (2011) New adapiform primate of Old World affinities from the Devil’s Graveyard Formation of Texas. Journal of Human Evolution 61: 156–168. 21571354 10.1016/j.jhevol.2011.02.014

[103] Tsukui K, Flynn JJ, Ramezani J, Machlus M, Bowring S (2013) Temporal calibration of the Bridgerian North American Land Mammal Age (NALMA): magnetostratigraphy and high precision U-Pb zircon geochronology of the middle Eocene Bridger Formation, Wyoming. Journal of Vertebrate Paleontology, Program and Abstracts, 2013, 228.

